# Improving Statistical Certainty of Glycosylation Similarity between Influenza A Virus Variants Using Data-Independent Acquisition Mass Spectrometry

**DOI:** 10.1016/j.mcpro.2022.100412

**Published:** 2022-09-11

**Authors:** Deborah Chang, Joshua Klein, William E. Hackett, Mary Rachel Nalehua, Xiu-Feng Wan, Joseph Zaia

**Affiliations:** 1 Department of Biochemistry, Center for Biomedical Mass Spectrometry, Boston University School of Medicine, Boston, Massachusetts, USA; 2 Boston University Bioinformatics Program, Boston University, Boston, Massachusetts, USA; 3 Center for Influenza and Emerging Infectious Diseases, University of Missouri, Columbia, Missouri, USA; 4 Department of Molecular Microbiology and Immunology, School of Medicine, University of Missouri, Columbia, Missouri, USA; 5 Department of Electrical Engineering & Computer Science, College of Engineering, University of Missouri, Columbia, Missouri, USA; 6 Bond Life Sciences Center, University of Missouri, Columbia, Missouri, USA

**Keywords:** mass spectrometry, DIA, glycoproteomics, glycosylation similarity, influenza, hemagglutinin, AGC, automatic gain control, AGP, alpha-1-acid glycoprotein, DDA, data-dependent acquisition, DIA, data-independent acquisition, FDR, false discovery rate, HA, hemagglutinin, IAV, influenza A virus, MDCK, Madin–Darby canine kidney, MS, mass spectrometry, NCE, normalized collision energy, TIC, total ion chromatogram

## Abstract

Amino acid sequences of immunodominant domains of hemagglutinin (HA) on the surface of influenza A virus (IAV) evolve rapidly, producing viral variants. HA mediates receptor recognition, binding and cell entry, and serves as the target for IAV vaccines. Glycosylation, a post-translational modification that places large branched polysaccharide molecules on proteins, can modulate the function of HA and shield antigenic regions allowing for viral evasion from immune responses. Our previous work showed that subtle changes in the HA protein sequence can have a measurable change in glycosylation. Thus, being able to quantitatively measure glycosylation changes in variants is critical for understanding how HA function may change throughout viral evolution. Moreover, understanding quantitatively how the choice of viral expression systems affects glycosylation can help in the process of vaccine design and manufacture. Although IAV vaccines are most commonly expressed in chicken eggs, cell-based vaccines have many advantages, and the adoption of more cell-based vaccines would be an important step in mitigating seasonal influenza and protecting against future pandemics. Here, we have investigated the use of data-independent acquisition (DIA) mass spectrometry for quantitative glycoproteomics. We found that DIA improved the sensitivity of glycopeptide detection for four variants of A/Switzerland/9715293/2013 (H3N2): WT and mutant, each expressed in embryonated chicken eggs and Madin–Darby canine kidney cells. We used the Tanimoto similarity metric to quantify changes in glycosylation between WT and mutant and between egg-expressed and cell-expressed virus. Our DIA site-specific glycosylation similarity comparison of WT and mutant expressed in eggs confirmed our previous analysis while achieving greater depth of coverage. We found that sequence variations and changing viral expression systems affected distinct glycosylation sites of HA. Our methods can be applied to track glycosylation changes in circulating IAV variants to bolster genomic surveillance already being done, for a more complete understanding of IAV evolution.

## Highlights

• Need for complete sampling of glycopeptide glycoforms.
• Comparison of DDA *versus* DIA for glycoproteomics.
• DIA achieves deeper glycopeptide sampling than DDA.
• Best DIA performance for relatively pure target glycoproteins.

## Graphical Abstract

**Figure:**
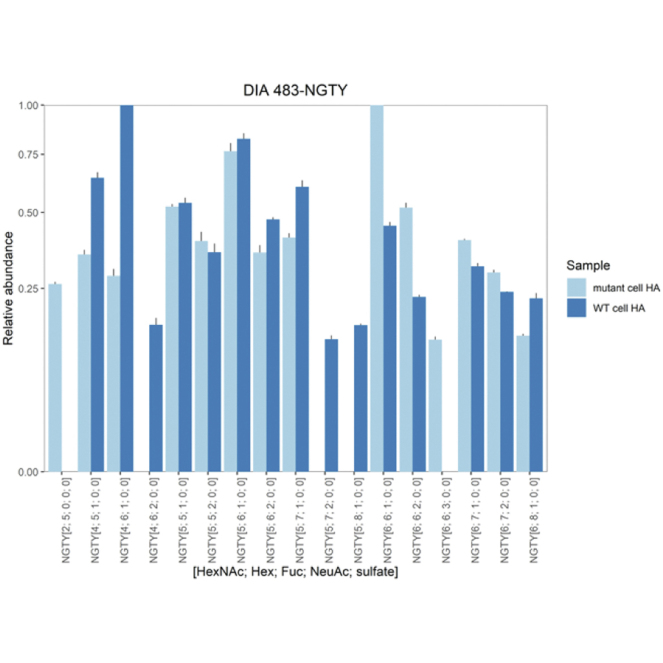

## In Brief

It is necessary to sample glycoprotein glycoforms deeply for biological studies. DDA is limited by missing values that result from limited scan speed and stochastic precursor ion selection. DIA samples all glycopeptide glycoforms, but duty cycle is reduced compared with DDA. DIA outperforms DDA for confident assignment of glycopeptides provided target glycoproteins are relatively pure.

Influenza A virus (IAV) undergoes antigenic drift and shift, respectively, bringing about evasion of immunity by seasonal strains and the emergence of novel strains against which humans have little immunity (1, 2). The need for pandemic preparedness has never been clearer, and global surveillance of seasonal and emerging IAV strains continues to be a major public health concern. Glycosylation of the envelope protein hemagglutinin (HA) of IAV has been shown to affect viral antigenicity and immunogenicity (3, 4). HA is the main avenue for interaction between IAV and host cells, and HA glycans may modulate receptor binding and allow for evasion of immune responses (5). Therefore, the global surveillance of IAV, which currently only involves genome sequencing, should include detailed and quantitative assessment of glycosylation changes on IAV glycoproteins as well.

It is important to maintain and improve annual vaccine readiness for the seasonal flu, especially because of the potential for the emergence of new epidemic strains. Most IAV vaccine production uses embryonated chicken eggs, but this greater than 70-year-old technology (6) has many drawbacks. The manufacturing process requires a large supply of high-quality eggs. Each dose of an inactivated vaccine requires three or four eggs for trivalent and quadrivalent vaccines, respectively. Scaling up production in a short amount of time, such as at the onset of an influenza pandemic, requires 6 to 12 months of lead time to build up chicken flocks. Extensive manufacturing protocols are needed to test for the microbial burden in eggs, and potential exposure of chicken flocks to avian influenza could further restrict egg supply (7). Furthermore, egg-expressed viruses exhibit adaptive mutations in the HA receptor–binding domain to enable binding to avian cellular receptors and infect chicken cells. The changes in receptor binding and antigenicity of these egg-adapted viruses have resulted in decreased vaccine effectiveness (8).

The World Health Organization has highlighted the urgent need for pursuing cell culture as an alternative means of production to increase the availability of influenza vaccine (9). The one mammalian cell–based inactivated influenza vaccine licensed for use by Food and Drug Administration virus grown in Madin–Darby canine kidney (MDCK) cells (https://www.cdc.gov/flu/prevent/cell-based.htm). Such mammalian cell lines hold advantages over egg-based production. Large stocks of MDCK cells may be frozen and banked in anticipation of pandemic strain emergence. MDCK cells circumvent the problem of egg adaptation. Amino acid sequence of receptor binding and antigenic regions of virus passaged in MDCK culture were found to be identical to those of clinical isolates, whereas egg-grown virus exhibited key mutations in those regions (10). In the 2017 to 2018 influenza season, cell culture–derived vaccine was observed to have a higher relative vaccine effectiveness than the egg-based vaccine among individuals aged 65 years and older (11). Furthermore, as noted previously, glycosylation plays an important role in antigenic shielding and could affect the immunodominance of antigenic sites. Therefore, quantitative comparisons of glycosylation between the two expression systems would benefit the efforts to improve global surveillance efforts of vaccine efficacy.

Our previous work (12) using data-dependent acquisition (DDA) mass spectrometry (MS) showed measurable glycosylation changes among IAV variants, even when the amino acid substitutions did not affect glycosylation sequons. DDA is a popular method that is capable of quantitative site-specific glycoprotein characterization. However, precursor ion selection in DDA is stochastic, compromising run-to-run reproducibility, and is biased toward higher abundance glycopeptides, leaving lower abundance ones undersampled. Data-independent acquisition (DIA) is an alternative method that has the potential to reduce bias and increase sensitivity and selectivity compared with DDA. Recent work has shown that DIA can improve sensitivity and confident quantification for intact glycopeptides (13, 14, 15); however, because of the unique complexities of the characterization and quantification of glycopeptides, there remain challenges to overcome. *N*-linked glycopeptides generally have much lower abundances than unmodified peptides, with a potentially vast hetereogeneity of glycan structures at each site. In addition, glycopeptides that share the same peptide backbone sequence but have different glycans often overlap in reversed-phase chromatography, leading to coeluting precursors that make MS/MS spectra interpretation difficult. MS/MS spectra interpretation is further complicated by the ubiquitous presence of highly abundant oxonium fragment ions that are common to many glycopeptides. In this work, we enriched viral samples for glycopeptides prior to MS analysis to maximize the glycopeptide signal, we used a relatively small DIA scanning window size to minimize coeluting precursors, and we used a relatively high collision energy to encourage the appearance of more information-rich fragment ions. We determined that DIA performed better than DDA using a standard glycoprotein alpha-1-acid glycoprotein (AGP). We showed in the A/Philippines/2/1982 (Phil82) IAV sample that there were some specific glycosylation sites with low abundance glycopeptides for which DIA was not as sensitive as DDA. Using DIA, we quantified the site-specific glycosylation of HA from WT strain A/Switzerland/9715293/2013 (H3N2) (SWZ13) and a mutated variant of SWZ13, comparing chicken egg and MDCK cell expression systems. We recapitulated our previous work showing low similarity between the WT and mutant egg–expressed virus at the 165-NVTM glycosylation site (12). We determined that the difference at this site is less pronounced between the WT and mutant cell–expressed virus. We also observed in the egg *versus* cell comparisons, the 285-NGSI and 483-NGTY sites are highly similar and highly dissimilar, respectively.

## Experimental Procedures

We used purified human AGP (Sigma–Aldrich; catalog no.: G9885) and the A/Philippines/2/1982 (Phil82) strain of IAV as glycoprotein standards. Phil82 was expressed in chicken eggs and generously provided to us by Dr Kevan Hartshorn at Boston University School of Medicine. The A/Switzerland/9715293/2013 (H3N2) (SWZ13) viral samples, both WT and 5B8 mutant, were propagated in parallel in specific pathogen-free embryonated chicken eggs and MDCK CCL-34 cells (American Type Culture Collection). The protocol for generation of the influenza virus has been described elsewhere (12). The mutant HA sequence, herein referred to as strain 5B8, had three amino acid substitutions, Q132H, Y219S, and D225N, none of which disrupted any *N*-glycosylation sequons.

### Glycopeptide Sample Preparation

Virus samples were first sonicated in methanol to disrupt virions before denaturation and reduction in 100 mM ammonium bicarbonate (J.T. Baker), 2-2-2 trifluoroethanol (Sigma–Aldrich), and 200 mM DTT (Sigma–Aldrich) at 65 °C for 1 h. A solution of 200 mM iodoacetamide (Bio-Rad) was added to alkylate cysteine residues in the dark for 1 h at room temperature. Additional DTT was added to the mixture to quench the alkylation reaction. A 3:1 mixture of water/100 mM ammonium bicarbonate was added to dilute the 2-2-2 trifluoroethanol to 5% by volume. Sequencing-grade trypsin and chymotrypsin (Promega) were applied in parallel reactions at an enzyme:substrate ratio 1:20 to digest the proteins into peptides, incubating at 37 °C overnight. The samples were heated to 95 °C for 10 min to inactivate the enzymes. Glycopeptides were enriched using iSPE-HILIC solid-phase extraction cartridges (HILICON) according to the manufacturer’s instructions. The enriched AGP glycopeptides were treated with α2-3,6,8 neuraminidase (New England Biolabs) to remove their terminal sialic acid residues. These glycopeptides are referred to as asialo AGP. A small aliquot from all samples was deglycosylated by incubating with 500 units of PNGase F (New England Biolabs) per 20 μg of glycoprotein at 37 °C overnight. As a final step, every sample was cleaned using PepClean C18 spin columns (Pierce Biotechnology). Prior to LC–MS/MS acquisition, 30 fmol of peptide retention time calibration mixture (Pierce Biotechnology; catalog no.: 88321) was spiked into each sample.

### LC–MS/MS Acquisition

All MS analyses were conducted on a Q Exactive HF instrument (Thermo Fisher Scientific) in positive mode. Chromatography was accomplished using a nanoAcquity UPLC, equipped with a nanoAcquity NPLC Symmetry C18 trap column, and an Acquity UPLC Peptide BEH C18 analytical column (Waters), heated to 37 °C, and a Triversa Nanomate source (Advion). We used 1% acetonitrile/0.1% formic acid for mobile phase A and 99% acetonitrile/0.1% formic acid for mobile phase B. We trapped analytes at 4 μl/min for 4 min in mobile phase A. Separation was done at 0.5 μl/min flow rate using the following gradient: 2 to 5% mobile phase B over 0 to 3 min, 5 to 40% B over 3 to 93 min, 40% B over 93 to 98 min, 40 to 98% B over 98 to 100 min, 98% B over 100 to 105 min, 98 to 2% B over 105 to 106 min, and 2% B over 106 to 120 min.

#### DDSA

MS1 spectra were acquired with resolution of 60,000 at *m/z* 400, 1 microscan per spectrum, scan range *m/z* 350 to 1800, 64 ms maximum injection time, and automatic gain control (AGC) target of 3e6 (AGC target of 1e6 was used for deglycosylated peptides). Up to 20 precursor ions were selected for fragmentation per cycle. MS2 spectra were acquired with resolution 30,000 at *m/z* 400, 2 microscans, 64 ms maximum injection time, AGC target 2e5 (1e6 for deglycosylated peptides), isolation window of *m/z* 2.0, isolation offset of *m/z* 0.4, scan range *m/z* 200 to 2000, normalized collision energy (NCE) of 35 (NCE of 27 was used for deglycosylated peptides), and 3.1e5 intensity threshold. Unassigned and +1 charge states were excluded, and dynamic exclusion was set to 20 s. Profile data were recorded for MS1 and MS2.

#### DIA

MS1 spectra were acquired with resolution 60,000 at *m/z* 400, 1 microscan per spectrum, scan range *m/z* 350 to 1800, 32 ms maximum injection time, and AGC target of 3e6. For MS2, we used 50 nonoverlapping isolation windows, *m/z* 16 wide, to cover the range of *m/z* 800 to 1600. Spectra were acquired with resolution 30,000 at *m/z* 400, 2 microscans, 32 ms maximum injection time, AGC target 1e6, and NCE 35. Profile and centroid data were recorded for MS1 and MS2, respectively.

### Data Analysis

All raw files were first converted to mzML format (16) using MSConvert (17) from ProteoWizard (Proteome Software), version 3.0.11252 with no additional filters.

The deglycoproteomics samples were processed using the Peaks PTM Search module of Peaks Studio 8.5 (Bioinformatics Solutions) (18) to provide information about the proteome for each sample type. AGP was searched against the entire UniProt Swiss-Prot (19) database (release 2017_04) with *Homo sapiens* as the species taxonomy and 555,085 valid proteins. Databases for Phil82 and the SWZ13 variants consisted of HA and neuraminidase sequences from each specific strain and internal IAV protein sequences from A/Puerto Rico/8/1934 (PR8) appended to the host proteome, either *Gallus gallus* (UP000000539 9031; release 2017_06) or *Canis lupus familiaris* (UP000002254 9615; release 2017_06). The actual number of entries actually searched were 29,551 for Phil82 and 29,628 for SWZ13. FASTA files can be found in supplemental Figs. S1 and S2. We searched technical replicates together, with trypsin or chymotrypsin selected as the proteolytic enzyme, a maximum of three missed cleavages, and one nonspecific cleavage allowed. We specified a fixed modification of cysteine carbamidomethylation and variable modifications of arginine deamidation and methionine oxidation. The precursor ion (MS1) mass error tolerance was 10 ppm, and the fragment ion (MS/MS) error tolerance was 0.02 Da. We required a minimum of two unique peptides for protein identification. We accepted default score thresholds for accepting individual spectra. We used a target-decoy false discovery threshold of 1% at the peptide level. Peaks Studio search results were exported in mzIdentML (20) format.

DDA and DIA raw files were processed using the GlycReSoft search engine, version 0.4.5 (21). DDA preprocessed used default GlycReSoft parameters. For DIA, we first performed deisotoping and charge state deconvolution, then using DIA-Umpire methodology (22), we used retention time to limit MS2 spectra with coisolating precursor ions. Only precursors with an apex retention time within 0.6 min of the product apex time were used, to account for retention time shifts because of coisolated precursors with shared product ions. Using a Pearson correlation, we correlated the precursors with the product ions, retaining up to the top 20 coisolating precursors. For these precursors, MS2 spectra were duplicated to contain information for a single precursor while retaining all the product ion information. After the preprocessing step, subsequent GlycReSoft searching steps were the same for DDA and DIA files.

The glycopeptide search space was built from the proteomics mzIdentML file and a glycan search space that was generated combinatorially following these glycan composition rules and restrictions. The number of Hex residues was 3 to 10, the number of HexNAc residues was 2 to 9, the number of Fuc residues was 0 to 5, and the number of Neu5Ac residues was 0 to 4. The number of Fuc was required to be less than the number of HexNAc, and the number of HexNAc was required to be greater than the number of Neu5Ac + 1. In addition, ≤1 sulfation and ≤1 ammonium adduct were considered. The same glycomics search space was used for AGP and IAV samples. GlycReSoft weighted and reported the score toward the localization with the fewest missing ions using a parsimony filter.

We used a false discovery threshold of 0.05. GlycReSoft used a peptide-centric method for scoring glycopeptide-spectrum matches, building upon the previously used coverage-weighted binomial model (21). Because we used a relatively high single collision energy for fragmentation without calibration for specific precursor *m/z* and charge states, we did not observe peptide + Y ions consistently for all glycopeptides, producing low scores for spectra that were otherwise well matched. Therefore, we did not allow any peaks matching theoretical peptide + Y ions to be included in scoring.

The abundances of glycopeptides that passed the false discovery rate (FDR) threshold of 0.05 were quantified by aggregating the all MS1 peak areas associated with each glycopeptide identity. Peptides with varying cleavages or post-translational modifications that had the same glycoform were summed. If a glycosite was identified in both tryptic and chymotryptic digestions, we retained only the set with the highest average abundance across replicates. Only glycan compositions that were observed in at least two replicates were considered positive identifications.

### Tanimoto Similarity Analysis

A detailed discussion of the Tanimoto similarity metric in the context of glycoprotein glycosylation has been previously published (12). We used an R toolkit called RAMZIS (23) that calculates a modified Tanimoto coefficient to estimate the similarity of glycosylation between two experimental groups, **A** and **B** (*e.g.*, WT *versus* mutant, DDA *versus* DIA, etc.). The comparisons account for the abundance of glycoforms observed as well as the proportion of the technical replicates in which the glycoforms were observed. A Tanimoto coefficient of 1 describes systems that share perfect similarity; a coefficient of 0 means the two groups are perfectly dissimilar. To represent the similarity and statistical confidence visually, we plot a “null” distribution composed of Tanimoto coefficients comparing randomly selected combinations of replicates from **A** and **B** combined. A “test” distribution of Tanimoto coefficients comparing randomly selected combinations of replicates from **A** to those of **B** is plotted with the null distribution. The overlap of the null and test distributions reflect the sum of the false positive (α) and false negative (β, or equivalently, 1 – power) rates. Thus, if we require a significance level of 0.05 (α = 5% of the joint area of both the null and test distributions) and 80% power (β = 20% of the joint area), then an overlap area of ≤25% indicates that we have statistically significant evidence to distinguish false positives and that the data had sufficient power to make a statistically confident conclusion about the similarity of the two samples (12). The internal quality plots (supplemental Figs. S7 and S8) display internal distributions, composed of Tanimoto coefficients calculated from combinations of replicates within a single sample. RAMZIS uses a few key metrics to evaluate the dataset. The internal quality of the dataset is measured by the confidence score of the internal similarity distributions. This score is relative to the comparison and should be above 2 to reliably distinguish changes in the glycosylation patterns. The observed similarity (*i.e.*, the test similarity between the original unbootstrapped samples) should be within the central quartiles (25–75%) of the test similarity distribution. This ensures that RAMZIS is accurately simulating the comparison. As a secondary fail safe to prevent spurious differentiation, the overlap between the internal and test distributions should be less than 25% of their total joint area for any differentiable glycosite. Finally, a glycosylation distribution is only differentiable if it has passed the aforementioned quality tests and it sees that the test and null similarity distributions have minimal overlap; there should be less than 5% of area overlap in the false-positive region, in *gray*, where the null is greater than the test, and less than 20% of the false-negative region, in *black*, where the test is greater than the null (12, 24).

### Experimental Design and Statistical Rationale

We acquired three technical replicates for each enzyme-digested sample of AGP and four technical replicates for all Phil82 and SWZ13 enzymatic digestions. AGP and Phil82 were used as control samples. All replicates were randomized in the acquisition queue to minimize batch effect bias.

## Results

All Peaks Studio search results, including precursor charge, *m/z*, modifications, peptide identification score, accession numbers, and protein groups, from the deglycosylated proteomics samples can be found in supplemental File S1. GlycReSoft search results are also provided in supplemental File S1. Site-specific glycoform abundances for each replicate are provided in supplemental File S2. Site-specific glycoform abundances are only included in the supplemental File S2 if they were identified in at least two replicates. All MS raw data, as well as annotated spectra for all glycopeptides identified, have been deposited in the ProteomeXchange Consortium (http://proteomecentral.proteomexchange.org/) *via* the PRIDE partner repository with the dataset identifier PXD029700 and DOI 10.6019/PXD029700.

### Comparisons of DDA and DIA Using Glycoprotein Standard Samples, AGP and Phil82

**Fig. 1:**
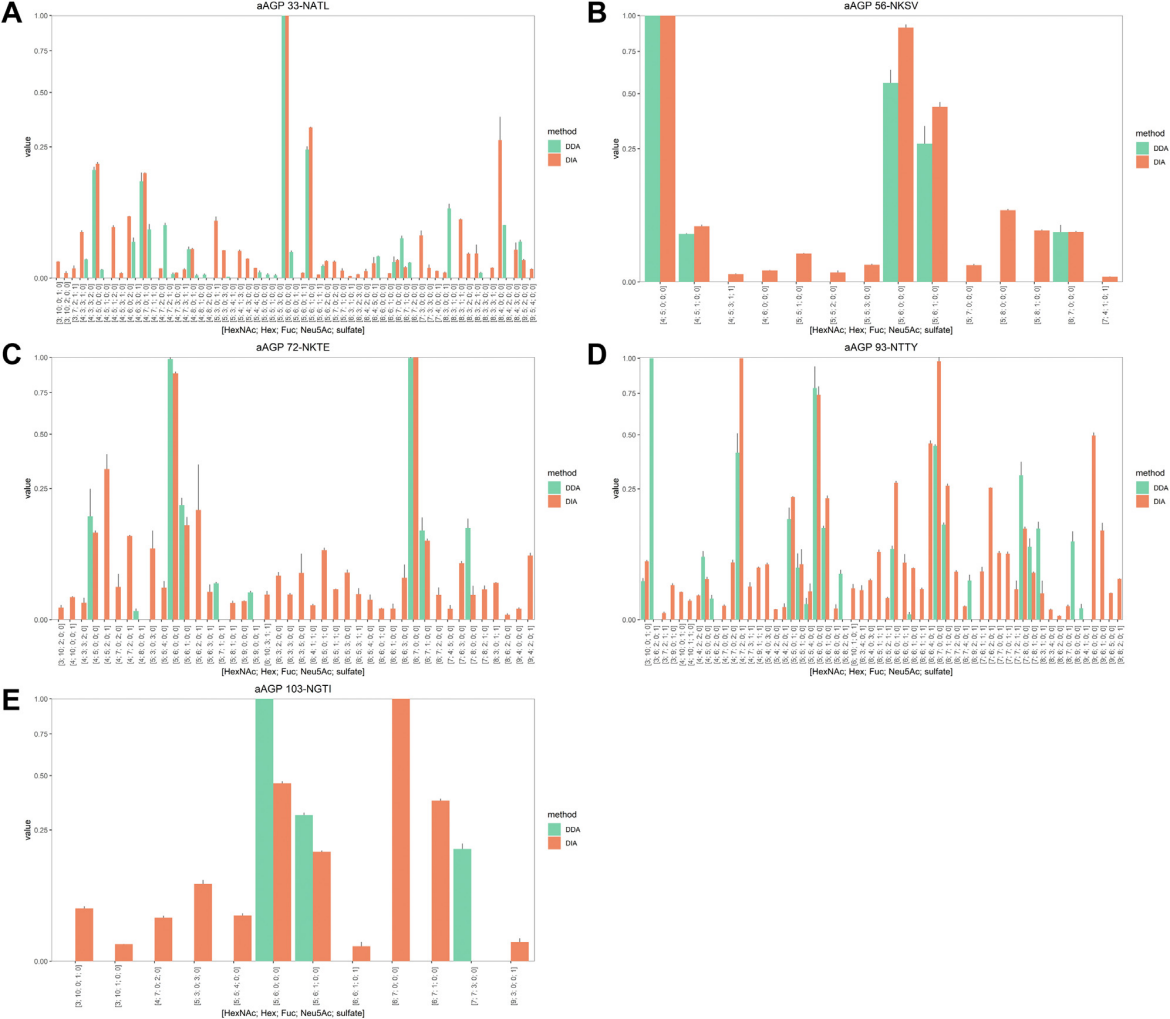
**Mean relative abundance of glycoforms of the A1AG1 isoform of AGP by DDA and DIA.** All five sites were observed: (*A*) 33-NATL, (*B*) 56-NKSV, (*C*) 72-NKTE, (*D*) 93-NTTY, and (*E*) 103-NGTI. Error bars indicate ±standard deviation. Glycoforms were required to have been observed in at least two replicates to be included. AGP, alpha-1-acid glycoprotein; DDA, data-dependent acquisition; DIA, data-independent acquisition.

**Fig. 2:**
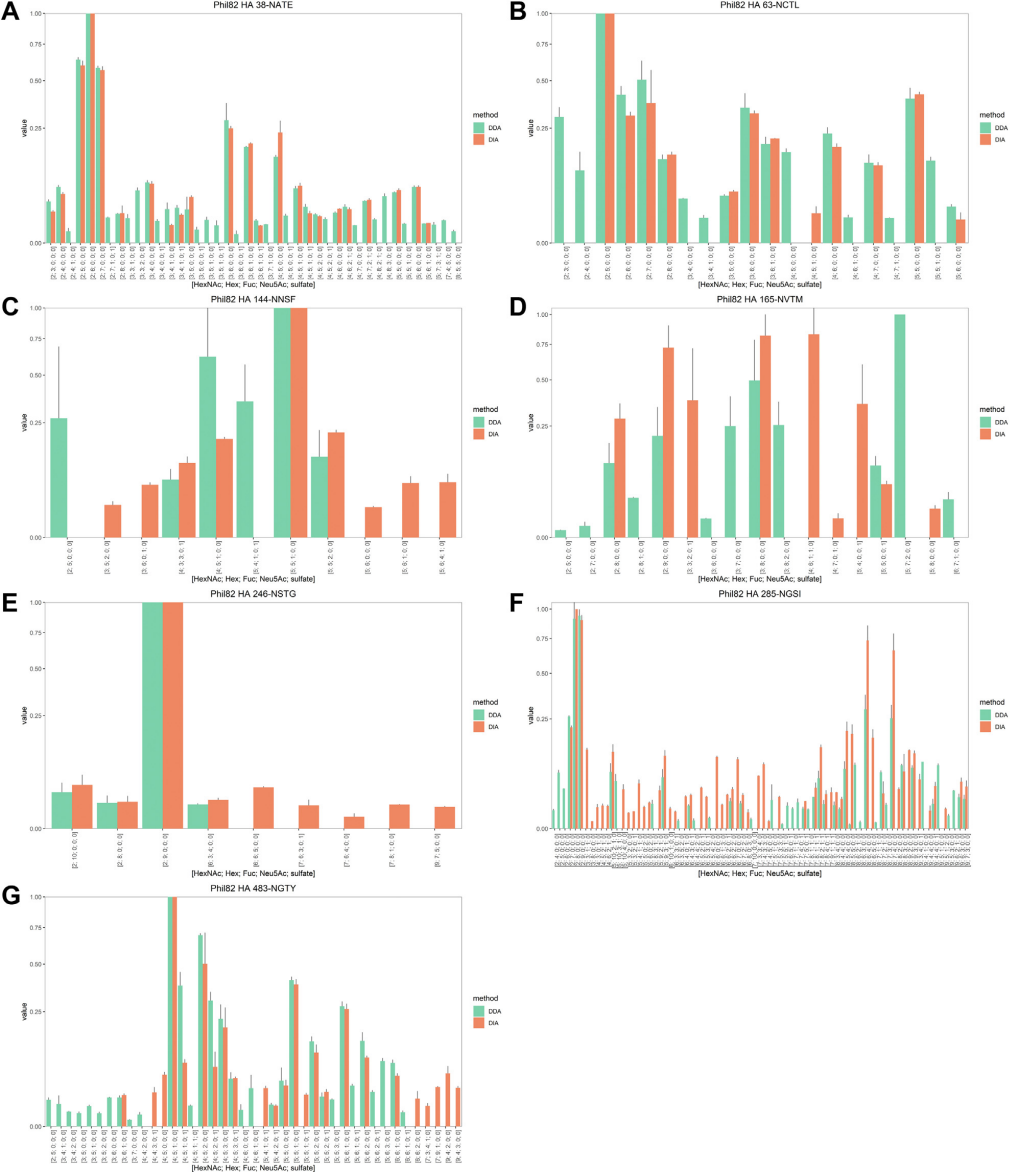
**Mean relative abundance of glycoforms of Phil82 HA by DDA and DIA.** Seven sites were observed: (*A*) 38-NATE, (*B*) 63-NCTL, (*C*) 144-NNSF, (*D*) 165-NVTM, (*E*) 246-NSTG, (*F*) 285-NGSI, and (*G*) 483-NGTY. Error bars indicate ±standard deviation. Glycoforms were required to have been observed in at least two replicates to be included. DDA, data-dependent acquisition; DIA, data-independent acquisition; HA, hemagglutinin.

Glycoforms from all five sites of the A1AG1 isoform of AGP were observed using both DDA and DIA. The number of glycoforms at each site that were observed in at least two of three replicates are shown in Table 1 and Figure 1. As shown in Figure 1, *A*–*E*, in all five glycosylation sites, DIA identified more glycoforms. Phil82 data are shown in Table 1 and Figure 2. As shown in Figure 2, *A*–*G*, of the 10 possible glycosylation sites of Phil82 HA, we observed three sites in which DIA yielded a larger number of glycoforms than DDA, 144-NNSF, 246-NSTG, and 285-NGSI, and four sites in which DIA underperformed DDA, 38-NATE, 63-NCTL, 165-NVTM, and 483-NGTY. The remaining three sites, 8-NSTA, 22-NGTL, and 126-NWTG, had too few observations to make any meaningful conclusions. Generally, higher abundance glycoforms were observed equally in both DDA and DIA. supplemental Fig. S3 displays density plots for the MS1 abundance for all precursors from all replicates and enzymatic digestions. supplemental Fig. S3, *A* and *B* displays the MS1 abundances for all glycopeptide precursors from the A1AG1 isoform of AGP (A) and Phil82 HA (B) meeting the GlycReSoft FDR threshold of 0.05, from all replicates and enzymatic digestions. The median abundances of DIA precursors were lower than those of DDA precursors.

**Table 1:** The number of glycoforms identified by DDA and DIA for asialo AGP and Phil82

Sample	Glycosite	# Glycoforms by DDA	# Glycoforms by DIA
AGP	33-NATL	27	44
	56-NKSV	5	14
	72-NKTE	9	39
	93-NTTY	21	50
	103-NGTI	3	11
	Total	65	158
Phil82	8-NSTA∗	0	1
	22-NGTL∗	0	0
	38-NATE	42	23
	63-NCTL	19	12
	126-NWTG∗	2	0
	144-NNSF	6	9
	165-NVTM	12	9
	246-NSTG	4	9
	285-NGSI	47	57
	483-NGTY	32	24
	Total	165	144

A glycoform was required to have been observed in at least two replicates to be included in this table. The sites with asterisks had too few observations to make meaningful conclusions.

### SWZ13 HA Variants

SWZ13 HA had 12 *N*-glycosylation sites, but we reliably detected glycoforms for only four sites, 8-NSTA, 165-NVTM, 285-NGSI, and 483-NGTY, across all four SWZ13 variants. The number of glycoforms observed in at least two replicates for these four sites, identified by each acquisition method, is shown in Table 2. (supplemental Table S1 shows the data for all other glycosylation sites.) supplemental Fig. S3, *C*–*F* displays density plots for the MS1 abundance for all precursors meeting the GlycReSoft FDR threshold of 0.05, from all replicates and enzymatic digestions. Just as in asialo AGP and Phil82 HA, the median abundances of DIA precursors were lower than those of DDA precursors.

**Table 2:** The number of glycoforms identified by DDA and DIA for the four variants of SWZ13 IAV for sites 8-NSTA, 165-NVTM, 285-NGSI, and 483-NGTY

Glycosite	WT egg		WT cell		Mutant egg		Mutant cell	
	DDA	DIA	DDA	DIA	DDA	DIA	DDA	DIA
8-NSTA	0	9	2	7	1	6	1	4
165-NVTM	9	11	5	6	8	9	9	7
285-NGSI	8	10	6	5	4	8	4	8
483-NGTY	15	18	12	15	7	8	11	14
Total	32	48	25	33	20	31	25	33

A glycoform was required to have been observed in at least two replicates to be included in this table. The number of glycoforms identified for all other sites can be found in supplemental Table S1.

In supplemental File S3, we compare annotated spectra for glycopeptides that were identified by DDA, but missed by DIA, showing the MS2 score of the matched DDA spectra to the expected score for the DIA spectra where the glycopeptide would have been found. In general, the DIA spectra had fewer b- and y-ions matching the expected fragment ions, resulting in lower MS2 scores than for DDA.

**Fig. 3:**
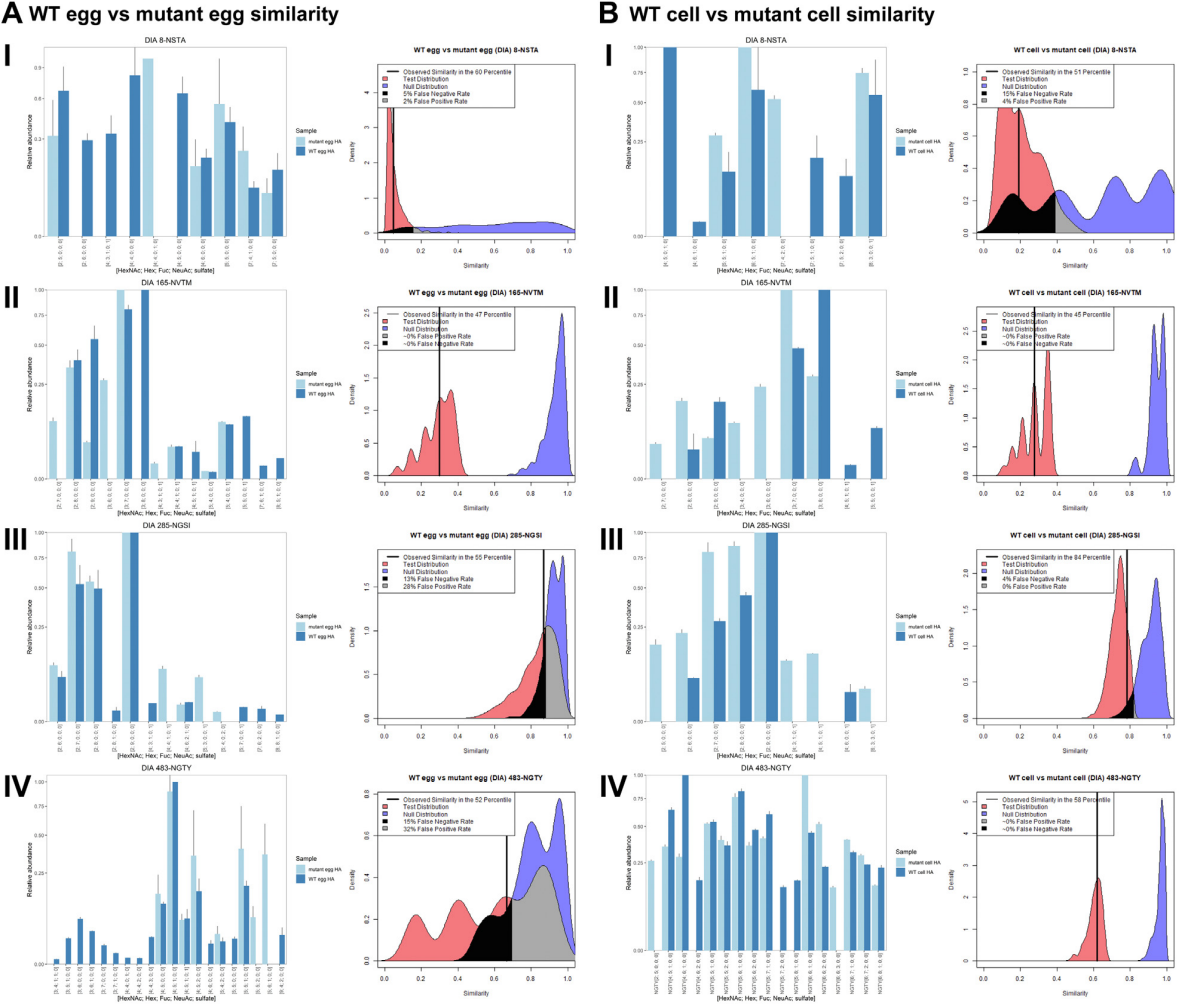
**Mean abundance and Tanimoto similarity for WT *versus* mutant SWZ13 HA.** The samples were derived from (*A*) embryonated chicken egg and (*B*) MDCK cell expression systems. The glycosylation sites shown are (i) 8-NSTA, (ii) 165-NVTM, (iii) 285-NGSI, and (iv) 483-NGTY. Bar plots display standardized mean abundances ±standard deviation. In the Tanimoto plots, the null distributions are shown in *blue*, the test distributions in *red*, and the observed similarities are represented by the *vertical black line*. HA, hemagglutinin; MDCK, Madin–Darby canine kidney.

**Fig. 4:**
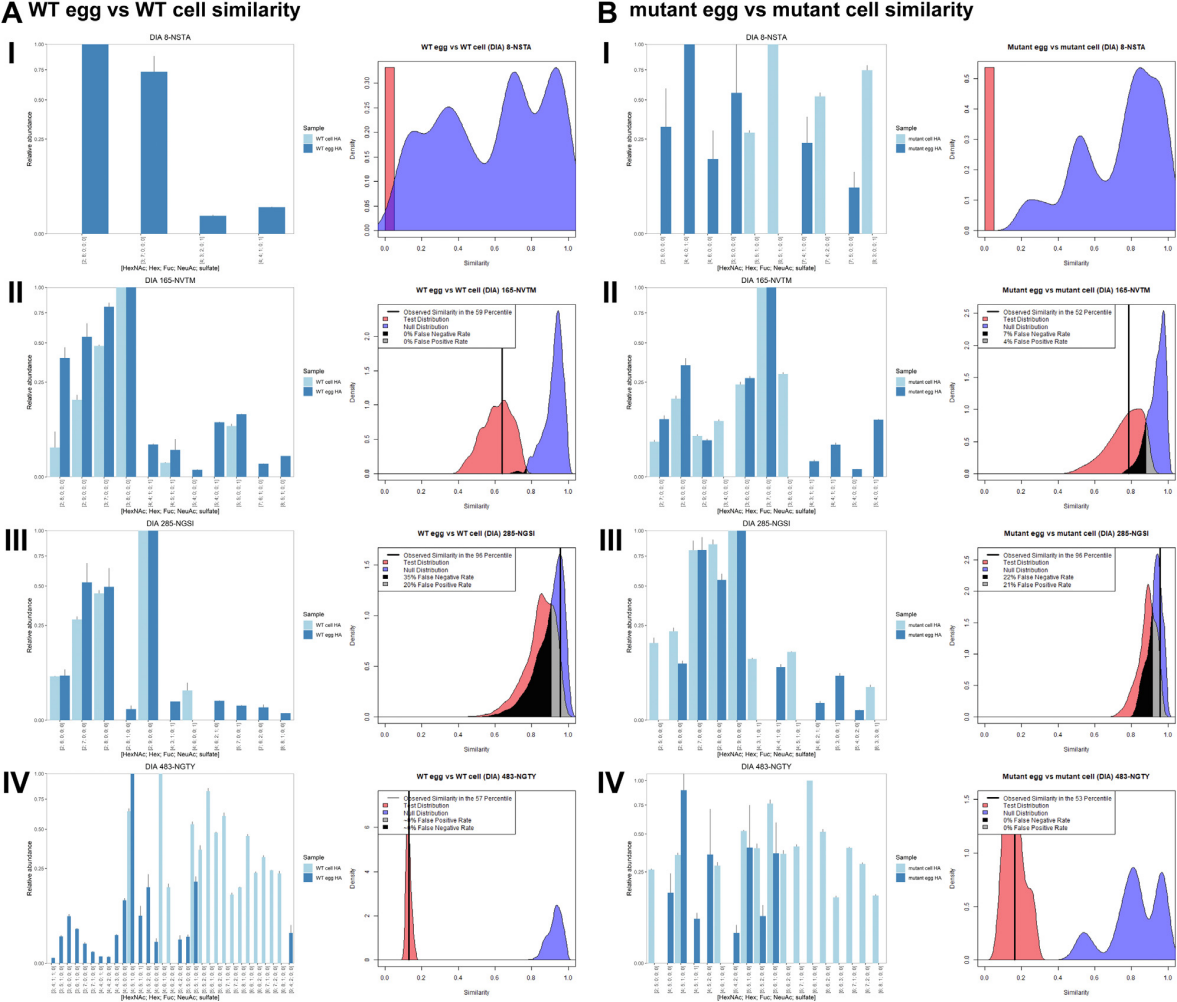
**Mean abundance and Tanimoto similarity for egg *versus* cell SWZ13 HA.** The samples were (*A*) WT and (*B*) mutant HA. The glycosylation sites shown are (i) 8-NSTA, (ii) 165-NVTM, (iii) 285-NGSI, and (iv) 483-NGTY. Bar plots display standardized mean abundances ±standard deviation. In the Tanimoto plots, the null distributions are shown in *blue*, the test distributions in *red*, and the observed similarities are represented by the *vertical black line*. HA, hemagglutinin.

We compared the four SWZ13 variants for the four glycosylation sites that were consistently in the following combinations: WT egg *versus* mutant egg (Fig. 3*A*), WT cell *versus* mutant cell (Fig. 3*B*), WT egg *versus* WT cell (Fig. 4*A*), and mutant egg *versus* mutant cell (Fig. 4*B*). The bar plots display the aggregated MS1 abundances for the glycoforms at each site by data acquisition method. The Tanimoto similarity plots were made using DIA-acquired data. Internal quality plots for each comparison can be found in supplemental Figs. S7 and S8. As described elsewhere (12), the internal quality plots are a quality control measure that provides guidance for how confident one can be in the dataset. In general, the green internal distributions in these figures represent the quality of the dataset for each experimental group; a tall and narrow internal distribution is considered better quality than a broad one. For example, site 8-NSTA had the broadest internal distributions, correlating to the fewest number of identifications as well. A summary of the Tanimoto similarities for all combinations is shown in Table 3.

**Table 3:** Tanimoto similarities for four glycosylation sites of SWZ13

Glycosite	Tanimoto similarity				IAV HA region
	WT egg *versus* mutant egg	WT cell *versus* mutant cell	WT egg *versus* WT cell	Mutant egg *versus* mutant cell	
8-NSTA	0.05	0.19	0	0	Stalk
165-NVTM	0.30	0.28	0.64	0.79	Head
285-NGSI	0.87	0.78	0.96	0.96	Stalk
483-NGTY	0.67	0.62	0.13	0.16	Stalk

These four sites were consistently observed across all four variants of SWZ13. The similarities for 8-NSTA were inconclusive because there were too few glycoforms observed to make confident comparisons.

## Discussion

### Comparing DIA and DDA Performance

Although we found that DIA was overall more sensitive than DDA, this was not always the case, and we learned that weighing the merits of both methods requires some nuanced thought. DIA is dependent on fast scanning speeds of instrument hardware, which are often not fast enough to achieve the sensitivity needed to identify all glycopeptides in a mixture, especially when the sample mixture is very complex and abundant. In cases where the sample is too complex and abundant, DIA fails to achieve its purported sensitivity. We found this to be especially true for Phil82, which had the most amount of glycopeptides injected on column compared with all the other samples. We had attempted to inject the same amount of material for all samples, but it is clear from supplemental Fig. S9, which shows total ion chromatograms (TICs) from selected tryptic digestions of each sample type, that Phil82 had the most complex TIC and highest TIC signal compared with the other sample types. AGP had approximately the same signal intensity, but AGP is less complex and does not have contaminating host proteins like an IAV sample does. The SWZ13 WT and mutant samples are potentially more complex than Phil82, having more glycosylation sites; however, it is clear that IAV protein concentrations that we started with in the digestion protocol were much smaller in all SWZ13 sample compared with Phil82. The TICs of the four SWZ13 samples had lower overall signal intensity and did not have many lower abundance glycopeptide peaks eluting as Phil82 did. This also explains why the SWZ13 samples had fewer overall glycopeptides identified than Phil82 did (Tables 1 and 2).

To better understand why DIA underperformed in Phil82, we parsed the raw data file from one DDA replicate of the tryptic Phil82 sample to extract instrument cycle information using RawTools (25). To calculate the time spent per scan, we divided the duty cycle by the number of MS2 fragmentation events triggered per cycle plus 1 (for the MS1 full scan). We obtained the same information for one DIA replicate of the tryptic Phil82 sample using the ms_deisotope tool from GlycReSoft. The time spent per scan for both DDA and DIA is shown in supplemental Fig. S4. Across the entire retention time range, the duty cycle, or time between MS1 scans, is longer for DIA than for DDA because DIA had more MS2 scans per cycle, 50 for DIA compared with ≤20 for DDA. The time spent per scan for DDA ranged from 160 to 250 ms. DIA is a fully deterministic method, with every cycle consisting of a full MS1 scan followed by 50 MS2 scans, so the time spent per scan stayed constant at approximately 155 ms across the time range. Thus, unlike for DDA, low abundance precursors did not have extra accumulation time, which would result in less intense fragment ions in the tandem mass spectra. Not only was DIA accessing precursors of lower abundance than DDA was (supplemental Fig. S3*B*), but the precursors that were identified by DDA but missed by DIA in the Phil82 samples had the lowest median abundance (supplemental Fig. S5). This is consistent with the product ion abundances of the glycopeptides from the four sites in which DIA underperformed. We compared the number and abundance of the b- and y-ions for DDA MS2 spectra that were positively identified *versus* those ions in the DIA MS2 spectra where they would have been identified but were missed (supplemental Fig. S6). We found that the b- and y-ions for these sites were fewer in number and less in abundance for the DIA spectra than for DDA. Our glycopeptide scoring algorithm was set to use solely b- and y-ion matches and, therefore, these sites had fewer identifications. In other words, the precursors that were missed by DIA had the lowest overall abundance, but the acquisition method could not adjust the accumulation time in the mass spectrometer, which explains why the fragment ions might be fewer and smaller.

AGP was a much less complex system, with fewer glycosylation sites, and the SWZ13 samples were lower in concentration at the outset, and for these samples, the sample complexity was sufficiently simple for DIA performance better than DDA. Thus, with the current hardware available to us, DIA would be a good choice to maximize sensitivity and quantification provided the sample matrix is not too complex. For relatively high concentration, high complexity samples with a background of host proteins, DIA alone would not be a good choice.

Supplemental File S3 displays annotated spectra for glycopeptides that were positively identified compared with the DIA spectra in which those same glycopeptide precursors would have appeared, if they had passed the scoring threshold.

### SWZ13 IAV Comparisons

The WT egg *versus* mutant egg comparison provides a confirmation of the conclusions from our previous work (12). This previous article, which used only DDA, showed that in egg-expressed viruses, there was a measurable difference in glycosylation between the WT and 5B8 mutant. The present work recapitulates the difference in quantification of the Man9GlcNAc2 glycoform at site 165-NVTM, with Man9GlcNAc2 having lower abundance in the mutant compared with WT. Also reproducible is the high similarity between WT and mutant for site 285-NGSI. Although the data for site 483-NGTY are less confident in the present work, nonetheless, we observed similar quantification for the most highly abundant glycoforms for this site. All these provide reassuring confirmation that that our analysis is reproducible.

Because of the sparseness of data for site 8-NSTA, we cannot conclude that this site is similar or dissimilar in any of the comparisons we made. However, the other three sites provide data with enough confidence for us to begin to understand how protein sequence changes or different viral expression systems may affect the glycosylation of the HA molecule. For site 165-NVTM, the egg-expressed virus had lower Man9GlcNAc2 in the mutant, recapitulating the results from our previous studies (12). This difference was present but less pronounced in the cell-grown viruses. The cell-grown viruses had the opposite quantification for Man8GlcNAc2, with a higher level of Man8GlcNAc2 in the mutant than in the WT. The similarity between the WT and mutant in the egg and cell systems for this site was both relatively low (0.30 and 0.28 in the egg and cell comparisons, respectively) because of these differences in Man8GlcNAc2 and Man9GlcNAc2 (Fig. 3, *A*ii, and *B*ii). These differences were no longer present in the egg *versus* cell comparisons (Fig. 4, *A*ii, and *B*ii), and consequently, the similarities were higher (0.64 and 0.74).

Site 285-NGSI had high similarity for all four comparisons (0.87 for WT egg *versus* mutant egg, 0.78 for WT cell *versus* mutant cell, and 0.96 for both WT egg *versus* WT cell and mutant egg *versus* mutant cell). However, for site 483-NGTY, the difference in glycosylation was much larger in the expression system comparison than for the sequence comparison. WT egg *versus* mutant egg and WT cell *versus* mutant cell yielded moderately high similarity values of 0.67 and 0.62, respectively. However, in both egg *versus* cell comparisons, we found very low similarity values of 0.13 and 0.16 in the WT and mutant, respectively. These low similarities can be attributed to the cell-expressed HA having larger complex-type glycans at this site, with more tri- and tetra-antennary glycans than in the egg-expressed HA. The 483-NGTY glycosylation site is found low in the stalk region of HA, away from the immunodominant receptor-binding domain and antigenic regions. Although the glycosylation state for this site is highly dissimilar in the two expression systems, it is likely that this site would not be involved in immune evasion or receptor binding. The three sites we compared (165-NVTM moderately high similarity, 285-NGSI high similarity, and 483-NGTY low similarity) are preliminary evidence that glycosylation would not affect the binding of antibodies against the HA head group but could conceivably alter binding of antibodies against the stalk.

## Conclusion

The glycoproteomics community strives for improving coverage and confidence in glycopeptide assignments, and DIA MS holds much promise in this area. Our work demonstrated that DIA improved the sensitivity of site-specific glycopeptide characterization in most cases, allowing for rigorous quantitative comparison of glycosylation similarity. However, more work is needed to reliably achieve the DIA promise of complete coverage and high confidence quantification for low abundance glycoforms. In this work, we used a relatively narrow DIA scanning window of *m/z* 16 windows but still found that spectra had contaminating fragment ions from coeluting precursors. Smaller scanning windows would reduce coelution at the expense of sensitivity. However, with instruments capable of faster scanning speeds, a better balance between specificity and sensitivity may be possible, especially for less complex samples. We also employed a single relatively high collision energy, which did not always produce high-abundance peptide + Y fragment ions in the tandem mass spectra, producing artificially low scores in what otherwise would have been well-matched spectra. Optimizing collision energies based on the characteristics of the glycopeptide analytes as well as developing new scoring algorithms should help to produce higher quality tandem mass spectra and to capture information for all glycopeptide fragment ion types, respectively.

## Data Availability

The MS data have been deposited to the ProteomeXchange Consortium (http://proteomecentral.proteomexchange.org/) *via* the PRIDE partner repository with the dataset identifier PXD029700 and DOI 10.6019/PXD029700.

RAMZIS and GlycReSoft may be freely downloaded at https://www.bumc.bu.edu/msr/software/.

## Supplemental data

This article contains supplemental data.

## Conflict of interest

The authors declare no competing interests.
