# Supplementary material for: Improving Statistical Certainty of Glycosylation Similarity between Influenza A Virus Variants Using Data-Independent Acquisition Mass Spectrometry
Source: Mol Cell Proteomics. 2022 Sep 11;21(11):100412. doi: 10.1016/j.mcpro.2022.100412 (PMC9593740; doi:10.1016/j.mcpro.2022.100412)
Supplement: supplementary_materials [file mmc1.docx]

**Improving statistical certainty of glycosylation similarity between influenza A virus variants using data-independent acquisition mass spectrometry**

**Authors:** Deborah Chang­, Joshua Klein, William E. Hackett, Mary Rachel Nalehua, Xiu-Feng Wan, Joseph Zaia

**List of materials:**

**Files**

- Supplemental File 1. Peaks Studio and GlycReSoft search results
  - Submitted as an Excel file (File1_peaks_studio_glycresoft_output.xlsx)
- Supplemental File 2. Glycopeptide abundances by replicate for each sample
  - Submitted as an Excel file (File2_glycopeptide_abundances_combined_replicates.xlsx)
- Supplemental File 3. Annotated spectra for glycopeptides that were identified by DDA but missed by DIA for SWZ13 WT and mutant
  - Submitted as four pdf files, zipped (File3_DDA_and_missed_DIA_annotated_spectra.zip)

**Figures**

- Figure S1. Hemagglutinin (HA) and neuraminidase (NA) sequences of A/Philippines/2/1982 virus.
- Figure S2. WT and 5B8 mutant hemagglutinin (HA) sequences, and neuraminidase (NA) sequence of A/Switzerland/9715293/2013 virus.
- Figure S3. Density plots for the MS1 abundance.
- Figure S4. Time spent per scan by acquisition method.
- Figure S5. Density plot for MS1 abundance of Phil82 precursors.
- Figure S6. Violin plots showing b- and y-ions from tandem mass spectra for Phil82 sites where DIA underperformed.
- Figure S7. Internal quality plots for site-specific comparisons of SWZ13 WT vs mutant.
- Figure S8. Internal quality plots for site-specific comparisons of SWZ13 egg vs cell.
- Figure S9. Total ion chromatograms for tryptic digestions of all samples.

**Tables**

- Table S1. The number of glycoforms identified by DDA and DIA for the four variants of SWZ13 IAV.

>iav|AFG99160|A/Phil/2/1982_HA A/Philippines/2/1982 1982// HA

MKTIIALSYMFCLVFAQNLPGNDNSTATLCLGHHAVPNGTLVKTITNDQIEVTNATELVQSSSTGRICDSPHRILDGKNCTLIDALLGDPHCDGFQNEKWDLFVERSKAFSNCYPYDVPDYASLRSLVASSGTLEFINEGFNWTGVTQSGGSSTCKRGSNNSFFSRLNWLYESESKYPVLNVTMPNNGKFDKLYIWGIHHPSTDKEQTNLYIRASGRVTVSTKRSQQTVIPNIGSRPWVRGLSSRISIYWTIVKPGDILLINSTGNLIAPRGYFKIRTGKSSIMRSDAPIGTCSSECITPNGSIPNDKPFQNVNKITYGACPRYVKQNTLKLATGMRNVPEKQTRGIFGAIAGFIENGWEGMVDGWYGFRHQNSEGTGQAADLKSTQAAIDQINGKLNRVIEKTNEKFHQIEKEFSEVEGRIQDLEKYVEDTKIDLWSYNAELLVALENQHTIDLTDSEMNKLFEKTRKQLRENAEDMGNGCFKIYHKCDNACIGSIRNGTYDHDVYRDEALNNRFQIKGVELKSGYKDWILWISFAISCFLLCVVLLGFIMWACQKGNIRCNICI

>iav|ADJ41819|A/Phil/2-MA/1982_NA A/Philippines/2-MA/1982 1982/06/01 NA

MNPNQKIITIGSVSLTIATICFLMQIAILVTTVTLHFKQYECSSPPNNQVVPCEPIIIERNITEIVYLTNTTIEKEICPKLVEYRNWSKPQCKITGFAPFSKDNSIRLSAGGDIWVTREPYVSCDPGKCYQFALGQGTTLDNKHSNDTIHDRTPYRTLLMNELGVPFHLGTRQVCIAWSSSSCHDGKAWLHVCITGYDKNATASFIYDGRLVDSIGSWSKNILRTQESECVCINGTCTVVMTDGSASERADTKILFIEEGKIVHISPLSGSAQHVEECSCYPRYPGVRCVCRDNWKGSNRPVVDINVKDYSIVSSYVCSGLVGDTPRKNDRSSSSYCRNPNNEKGNHGVKGWAFDDGNDVWMGRTISEESRSGYETFKVIGGWSTPNSKLQINRQVIVDSGNRSGYSGIFSVEGKSCINRCFYVELIRGREQETRVWWTSNSIVVFCGTSGTYGTGSWPDGADINLMPI

**Figure S1.** Hemagglutinin (HA) and neuraminidase (NA) sequences of A/Philippines/2/1982 virus.

>cus|SWZHA|A/Switzerland/9715293/2013 HA SWZ 132Q, 219Y, 225D 20190316

QKLPGNDNSTATLCLGHHAVPNGTIVKTITNDRIEVTNATELVQNSSIGEICDSPHQILDGENCTLIDALLGDPQCDGFQNKKWDLFVERSKAYSNCYPYDVPDYASLRSLVASSGTLEFNNESFNWAGVTQNGTSSSCRRGSNSSFFSRLNWLTHLNSKYPALNVTMPNNEQFDKLYIWGVHHPVTDKDQIFLYAQSSGRITVSTKRSQQAVIPNIGYRPRIRDIPSRISIYWTIVKPGDILLINSTGNLIAPRGYFKIRSGKSSIMRSDAPIGKCKSECITPNGSIPNDKPFQNVNRITYGACPRYVKQSTLKLATGMRNVPERQTRGIFGAIAGFIENGWEGMVDGWYGFRHQNSEGRGQAADLKSTQAAIDQINGKLNRLIGKTNEKFHQIEKEFSEVEGRIQDLEKYVEDTKIDLWSYNAELLVALENQHTIDLTDSEMNKLFEKTKKQLRENAEDMGNGCFKIYHKCDNACIGSIRNGTYDHDVYRDEALNNRFQIKGVELKSGYKDWILWISFAISCFLLCVALLGFIMWACQKGNIRCNICI

>cus|SWZHA_5B8|A/Switzerland/9715293/2013 HA 5B8 132H, 219S, 225N 20190316

QKLPGNDNSTATLCLGHHAVPNGTIVKTITNDRIEVTNATELVQNSSIGEICDSPHQILDGENCTLIDALLGDPQCDGFQNKKWDLFVERSKAYSNCYPYDVPDYASLRSLVASSGTLEFNNESFNWAGVTHNGTSSSCRRGSNSSFFSRLNWLTHLNSKYPALNVTMPNNEQFDKLYIWGVHHPVTDKDQIFLYAQSSGRITVSTKRSQQAVIPNIGSRPRIRNIPSRISIYWTIVKPGDILLINSTGNLIAPRGYFKIRSGKSSIMRSDAPIGKCKSECITPNGSIPNDKPFQNVNRITYGACPRYVKQSTLKLATGMRNVPERQTRGIFGAIAGFIENGWEGMVDGWYGFRHQNSEGRGQAADLKSTQAAIDQINGKLNRLIGKTNEKFHQIEKEFSEVEGRIQDLEKYVEDTKIDLWSYNAELLVALENQHTIDLTDSEMNKLFEKTKKQLRENAEDMGNGCFKIYHKCDNACIGSIRNGTYDHDVYRDEALNNRFQIKGVELKSGYKDWILWISFAISCFLLCVALLGFIMWACQKGNIRCNICI

>cus|SWZNA|A/Switzerland/9715293/2013 NA protein

MNPNQKIITIGSVSLTISTICFFMQIAILITTVTLHFKQYEFNSPPNNQVMLCEPTIIERNITEIVYLTNTTIEKEICPKPAEYRNWSKPQCGITGFAPFSKDNSIRLSAGGDIWVTREPYVSCDPDKCYQFALGQGTTLNNVHSNNTVRDRTPYRTLLMNELGVPFHLGTKQVCIAWSSSSCHDGKAWLHVCITGDDKNATASFIYNGRLVDSVVSWSKDILRTQESECVCINGTCTVVMTDGSASGKADTKILFIEEGKIVHTSTLSGSAQHVEECSCYPRYPGVRCVCRDNWKGSNRPIVDINIKDHSIVSSYVCSGLVGDTPRKNDSSSSSHCLDPNNEEGGHGVKGWAFDDGNDVWMGRTINETSRLGYETFKVIEGWSNPKSKLQTNRQVIVDRGDRSGYSGIFSVEGKSCINRCFYVELIRGRKEETEVLWTSNSIVVFCGTSGTYGTGSWPDGADLNLMPI

**Figure S2.** WT and 5B8 mutant hemagglutinin (HA) sequences, and neuraminidase (NA) sequence of A/Switzerland/9715293/2013 virus.

**
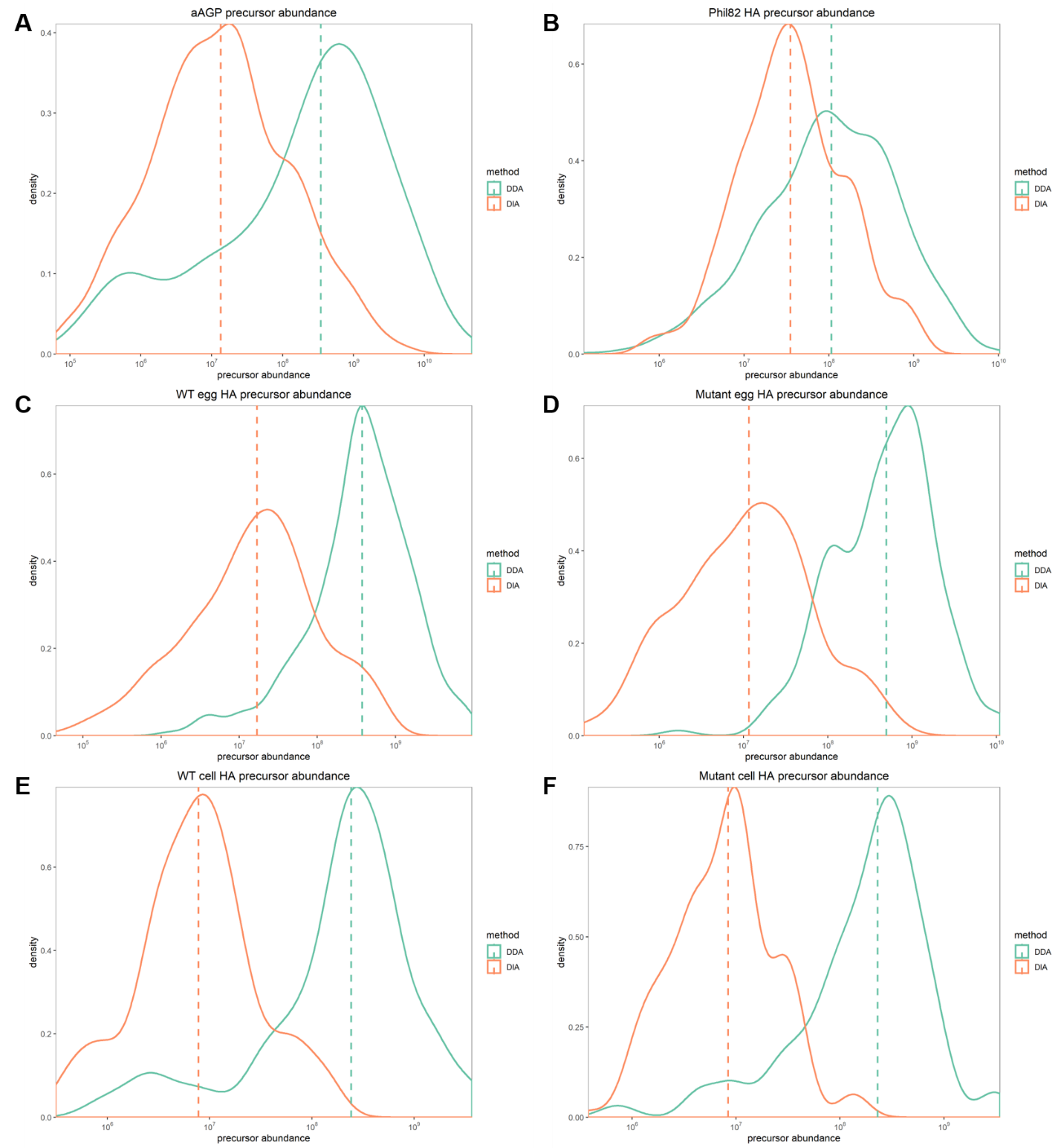
**

**Figure S3.** MS1 precursor abundance density plots. The MS1 abundances were for all precursors from all replicates and enzymatic digestions meeting the GlycReSoft FDR threshold of 0.05 by acquisition method. **(A)** Precursors from the A1AG1 isoform of AGP, **(B)** Phil82 HA, **(C)** WT SWZ13 HA expressed in egg, **(D)** mutant SWZ13 HA expressed in egg, **(E)** WT SWZ13 HA expressed in cell, and **(F)** mutant SWZ13 HA expressed in cell. Dashed vertical lines display the median abundances for each distribution.

**
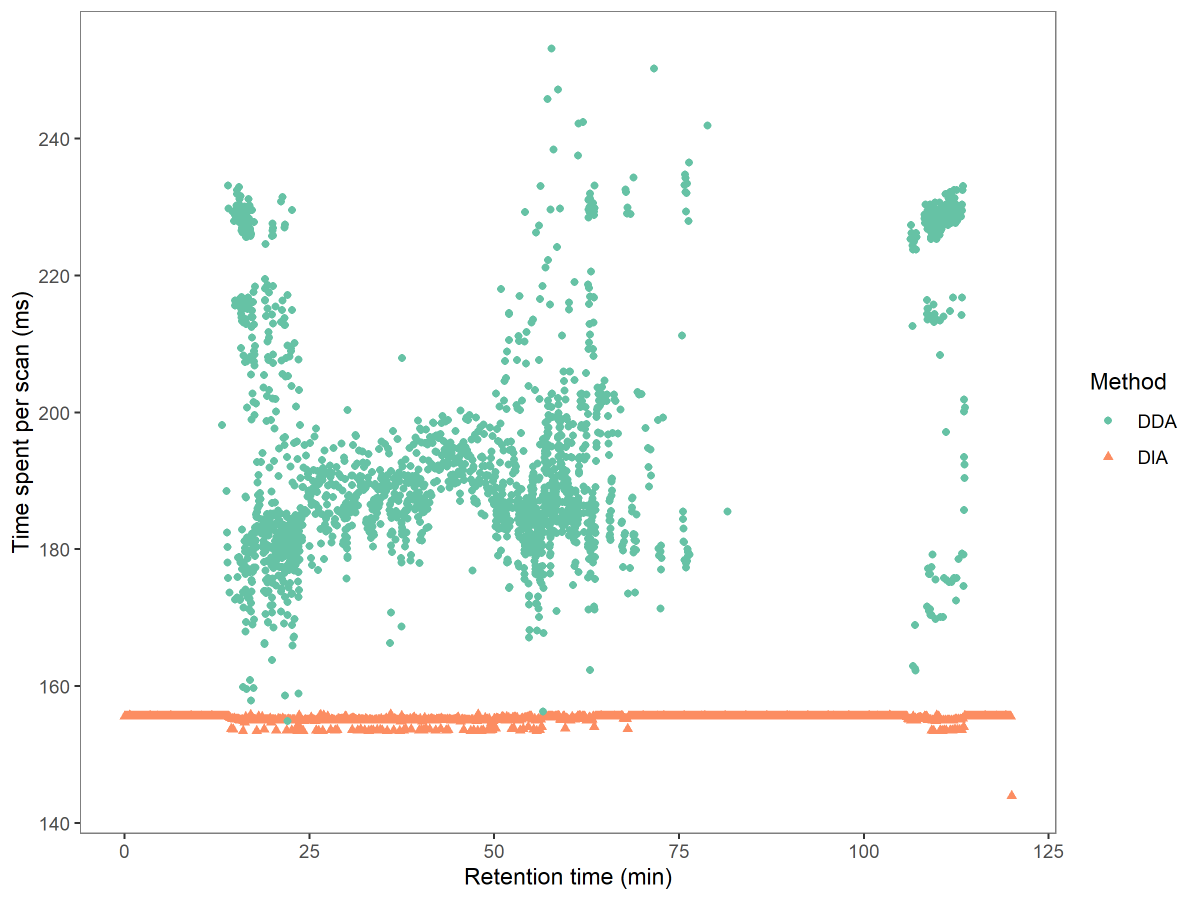
**

**Figure S4.** The time spent per scan for DDA and DIA. For each method, the duty cycle was divided by the number of MS2 scans triggered in each cycle + 1.

**
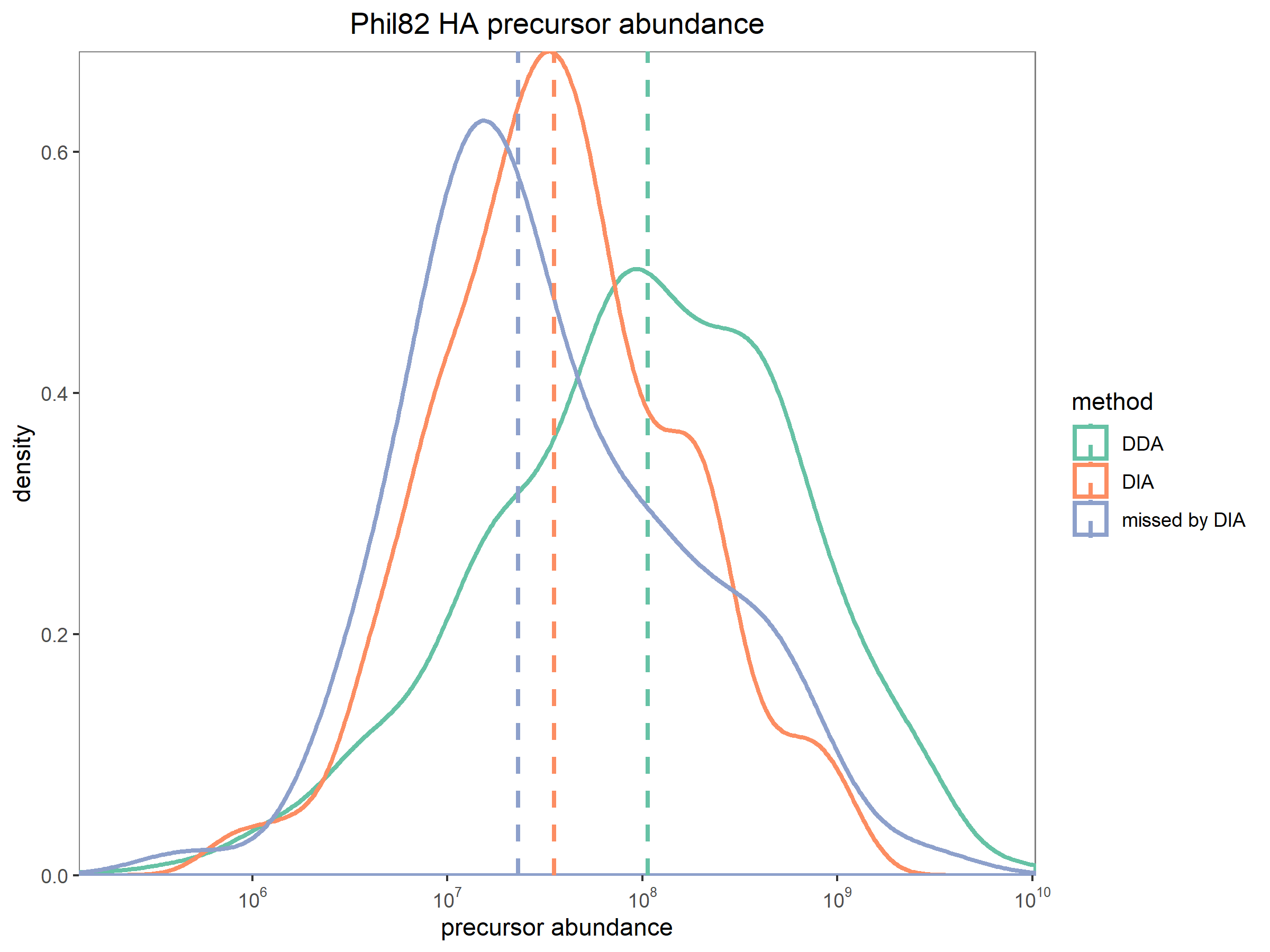
**

**Figure S5.** MS1 precursor abundance density plots for Phil82. The MS1 abundances were for all Phil82 precursors from all replicates and enzymatic digestions meeting the GlycReSoft FDR threshold of 0.05 by acquisition method. Dashed vertical lines display the median abundances for each distribution. The precursors for glycopeptides that were identified by DDA but not by DIA had the lowest median abundance over all.

**
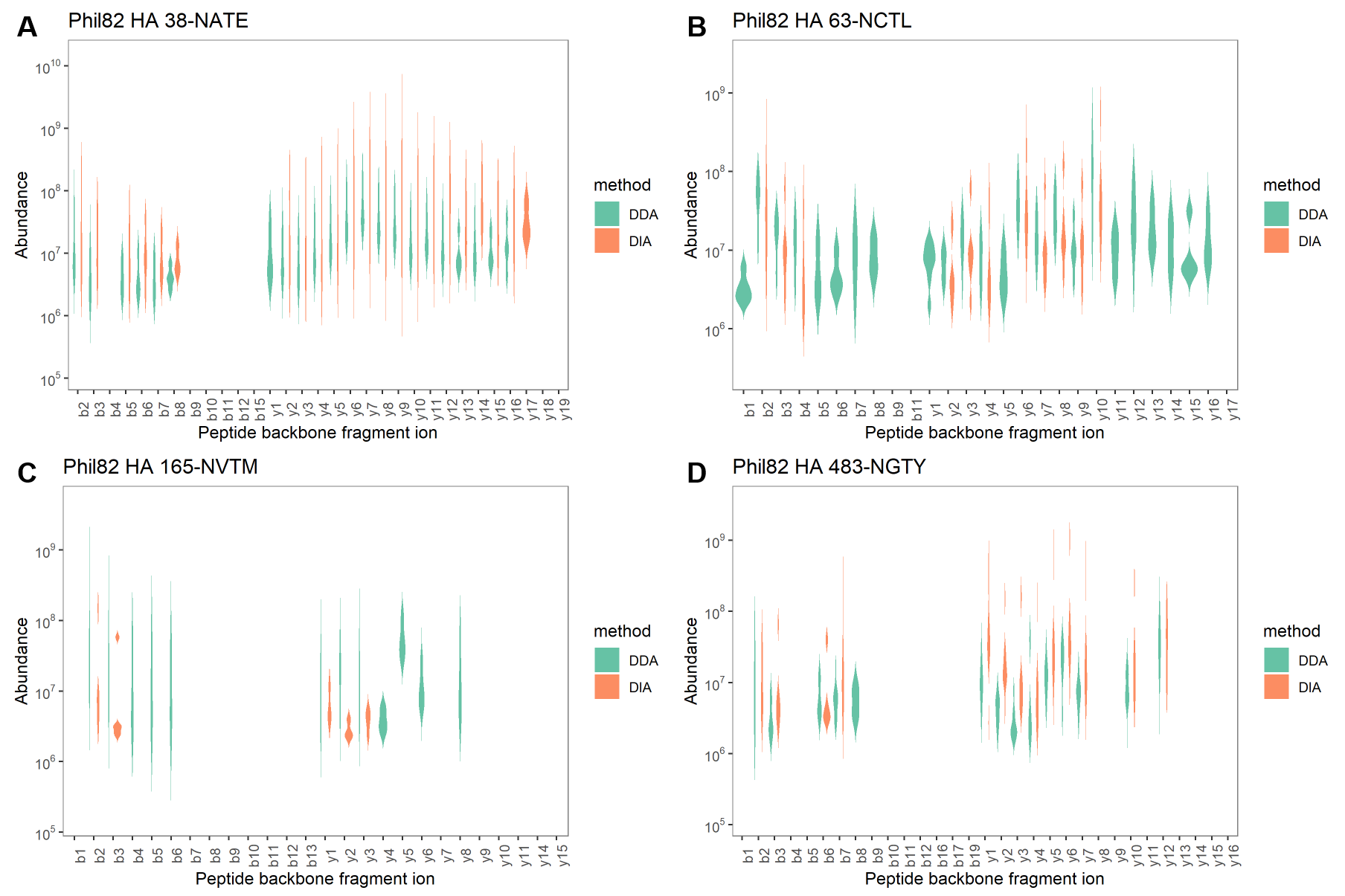
**

**Figure S6.** Abundance of b- and y-ions from Phil82 tandem mass spectra of glycopeptides assigned by DDA but not by DIA. The violin plots are for the following sites: (A) 38-NATE, (B) 63-NCTL, (C) 165-NVTM, and (D) 483-NGTY. The width of each violin is proportional to the number of that particular fragment ion type that matched the corresponding theoretical tandem mass spectra


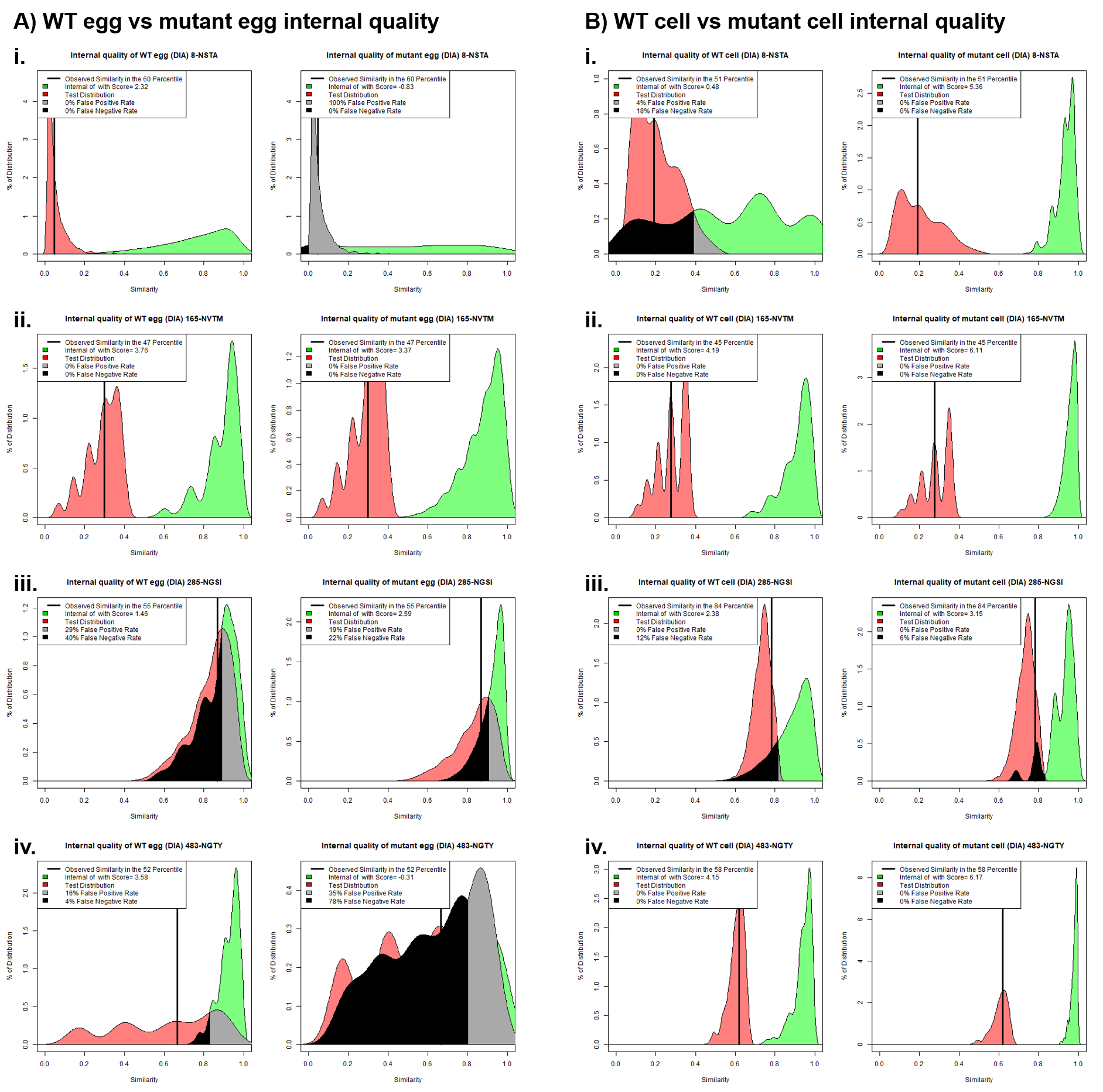


**Figure S7.** Internal quality for site-specific comparisons for SWZ13 variants. The similarity comparisons were: **(A)** WT egg vs mutant egg, and **(B)** WT cell vs mutant cell, corresponding to the plots in Figure 4. An internal distribution (green) is drawn for each experimental group in each comparison. **(i)** site 8-NSTA, **(ii)** site 165-NVTM, **(iii)** site 285-NGSI, and **(iv)** site 483-NGTY.


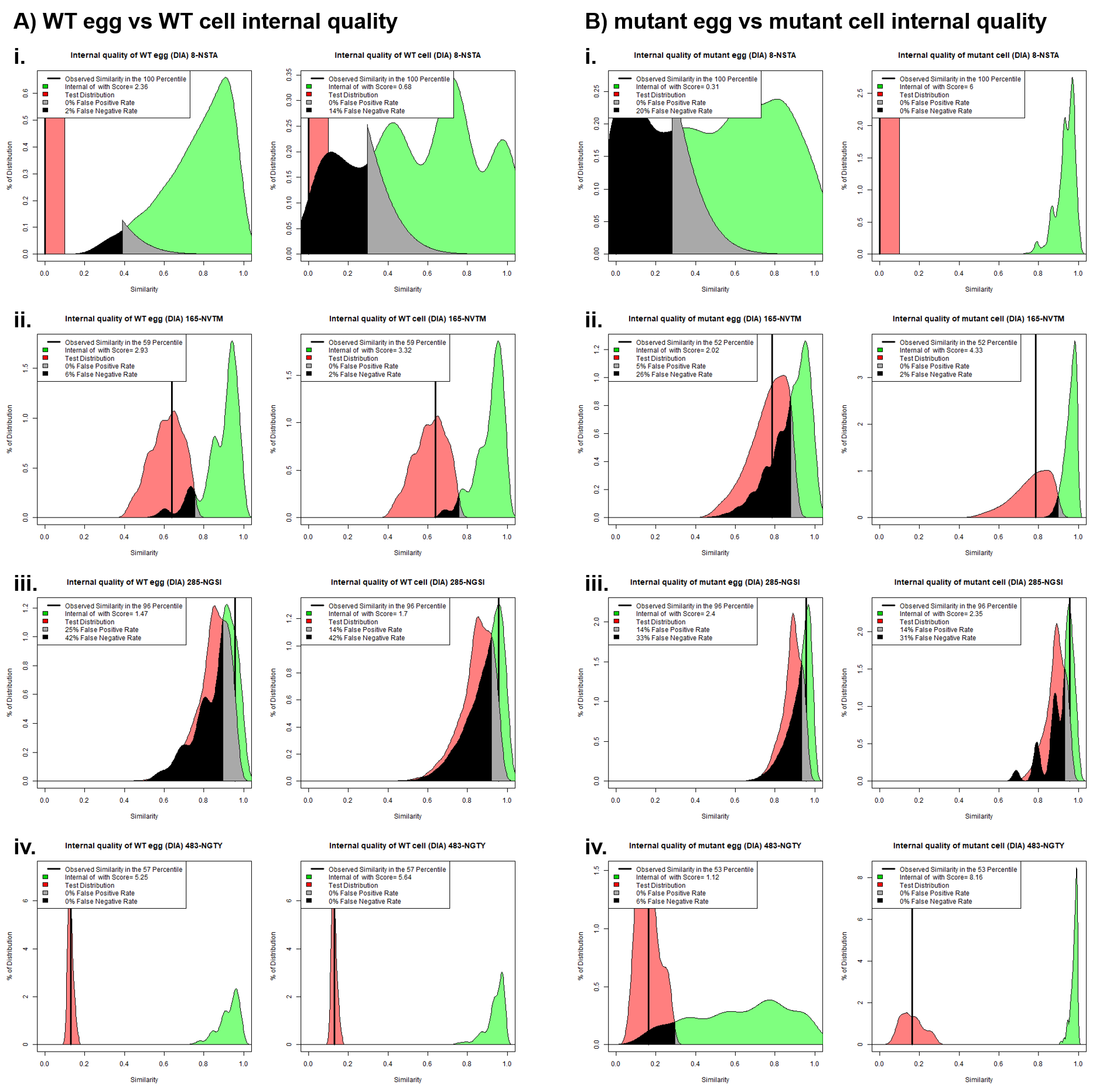


**Figure S8.** Internal quality for site-specific comparisons for SWZ13 variants. The similarity comparisons were: **(A)** WT egg vs WT cell, and **(B)** mutant egg vs mutant cell, corresponding to the plots in Figure 4. An internal distribution (green) is drawn for each experimental group in each comparison. **(i)** site 8-NSTA, **(ii)** site 165-NVTM, **(iii)** site 285-NGSI, and **(iv)** site 483-NGTY.


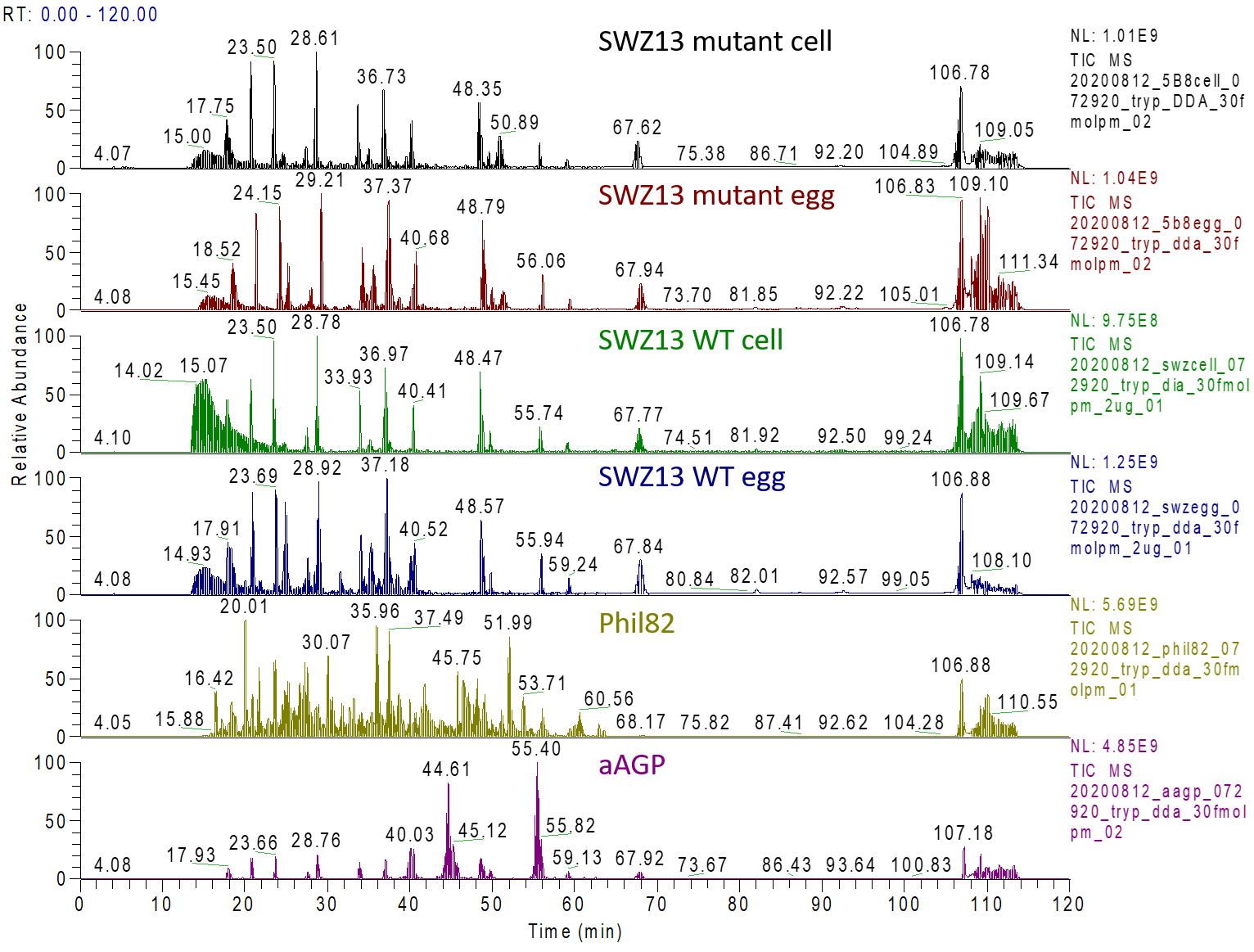


**Figure S9.** Total ion chromatograms (TIC) for tryptic digestions of all samples. We had attempted to inject the same amount of material for all samples, however, Phil82 had the most complex TIC and highest TIC signal compared to the other sample types.

Table S1. The number of glycoforms identified by DDA and DIA for the four variants of SWZ13 IAV. A glycoform was required to have been observed in at least two replicates to be included in this table. The sites with asterisks had too few observations to make meaningful conclusions.

|  | WT egg | | WT cell | | Mutant egg | | Mutant cell | |
| --- | --- | --- | --- | --- | --- | --- | --- | --- |
| Glycosite | DDA | DIA | DDA | DIA | DDA | DIA | DDA | DIA |
| 8-NSTA | 0 | 9 | 2 | 7 | 1 | 6 | 1 | 4 |
| 22-NGTI* | 0 | 4 | 0 | 0 | 0 | 0 | 0 | 0 |
| 38-NATE* | 1 | 2 | 0 | 0 | 0 | 0 | 0 | 0 |
| 45-NSSI* | 0 | 0 | 0 | 0 | 0 | 0 | 0 | 0 |
| 63-NCTL* | 0 | 0 | 0 | 0 | 0 | 0 | 0 | 0 |
| 122-NESF* | 0 | 0 | 0 | 0 | 0 | 0 | 0 | 0 |
| 133-NGTS* | 2 | 1 | 0 | 0 | 4 | 2 | 0 | 0 |
| 144-NSSF* | 5 | 7 | 0 | 0 | 3 | 4 | 1 | 3 |
| 165-NVTM | 9 | 11 | 5 | 6 | 8 | 9 | 9 | 7 |
| 246-NSTG* | 3 | 5 | 1 | 0 | 2 | 3 | 1 | 1 |
| 285-NGSI | 8 | 10 | 6 | 5 | 4 | 8 | 4 | 8 |
| 483-NGTY | 15 | 18 | 12 | 15 | 7 | 8 | 11 | 14 |
| Total | 43 | 67 | 26 | 33 | 29 | 40 | 27 | 37 |
