# Supplementary material for: Improving Statistical Certainty of Glycosylation Similarity between Influenza A Virus Variants Using Data-Independent Acquisition Mass Spectrometry
Source: Mol Cell Proteomics. 2022 Sep 11;21(11):100412. doi: 10.1016/j.mcpro.2022.100412 (PMC9593740; doi:10.1016/j.mcpro.2022.100412)
Supplement: File S3 [file mmc4.pdf]

Improving statistical certainty of glycosylation similarity between influenza A virus variants using data-independent acquisition mass spectrometry

Deborah Chang[1], Joshua Klein[2], William Hackett[2], Mary Rachel Nalehua[2], Xiu-Feng Wan[3,4,5,6], Joseph Zaia[1,2]

Supplementary File 3. Annotated tandem mass spectra for glycopeptides that were positively identified in the DDA data compared to spectra that failed to pass the scoring threshold in the DIA data.

SWZ mutant cell pp. 2-3

SWZ mutant egg pp. 4-7

SWZ wild type cell pp. 8-11

SWZ wild type egg pp. 12-21

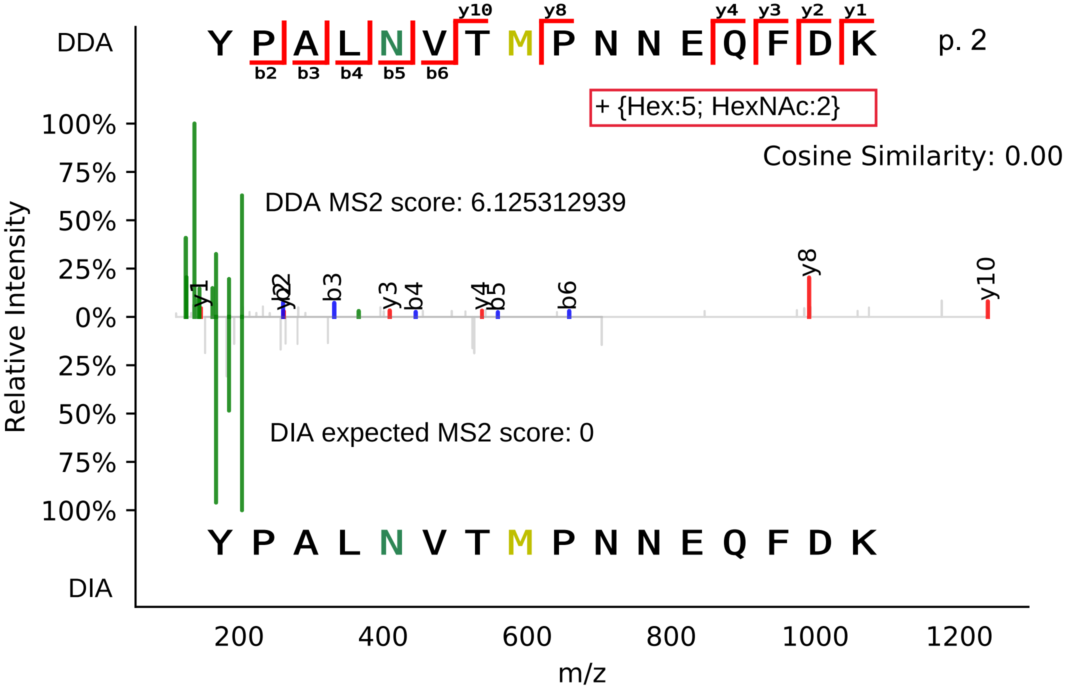
DDA
Y P A L N V T M P N N E Q F D K
y10 y8 y4 y3 y2 y1
b2 b3 b4 b5 b6
p. 2
+ {Hex:5; HexNAc:2}
Cosine Similarity: 0.00
DDA MS2 score: 6.125312939
DIA expected MS2 score: 0
Relative Intensity
100%
75%
50%
25%
0%
y1 b2 y2 b3 y3 b4 y4 b5 b6 y8 y10
Y P A L N V T M P N N E Q F D K
DIA
200 400 600 800 1000 1200
m/z

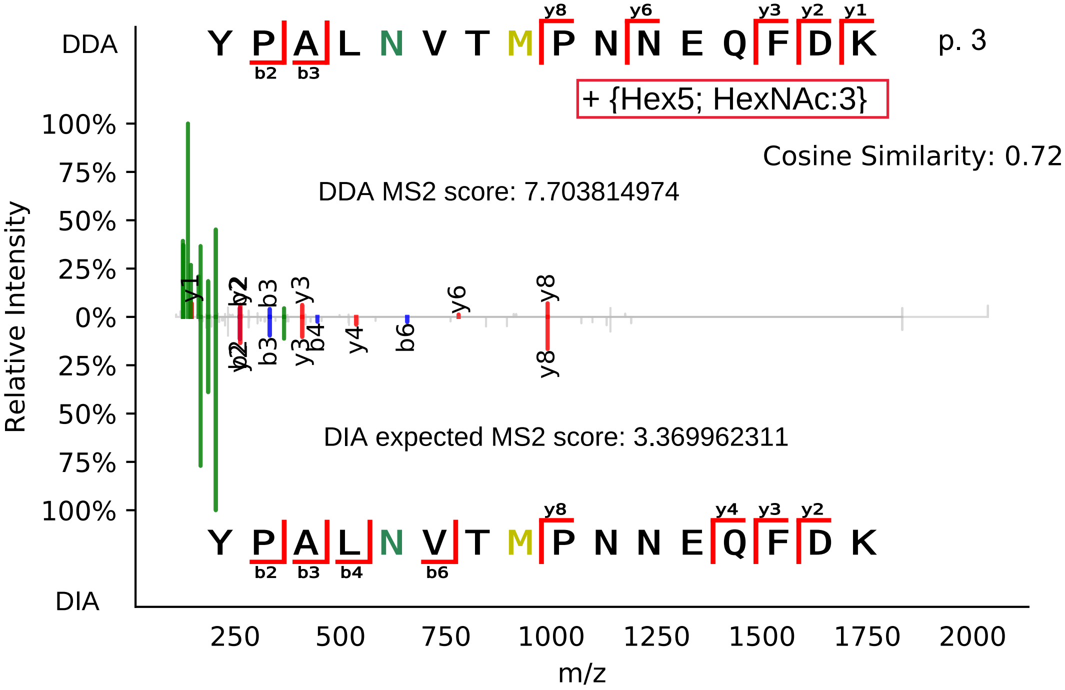
DDA
Y P A L N V T M P N N E Q F D K
y8 y6 y3 y2 y1
b2 b3
+ {Hex5; HexNAc:3}
p. 3
Cosine Similarity: 0.72
DDA MS2 score: 7.703814974
DIA expected MS2 score: 3.369962311
Relative Intensity
100%
75%
50%
25%
0%
y1
b2
y2
b3
y3
y6
y8
b4
y4
b6
Y P A L N V T M P N N E Q F D K
y8 y4 y3 y2
b2 b3 b4 b6
DIA
250
500
750
1000
1250
1500
1750
2000
m/z

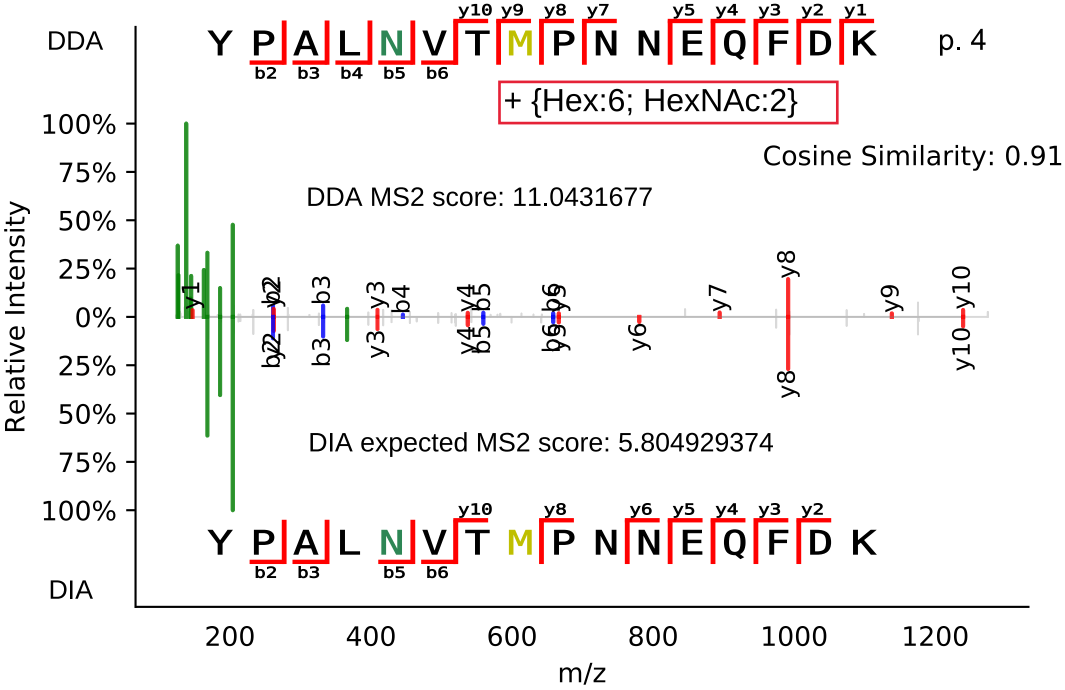
DDA
Y P A L N V T M P N N E Q F D K
p. 4
+ {Hex:6; HexNAc:2}
Cosine Similarity: 0.91
DDA MS2 score: 11.0431677
DIA expected MS2 score: 5.804929374
DIA
Y P A L N V T M P N N E Q F D K
Relative Intensity
m/z

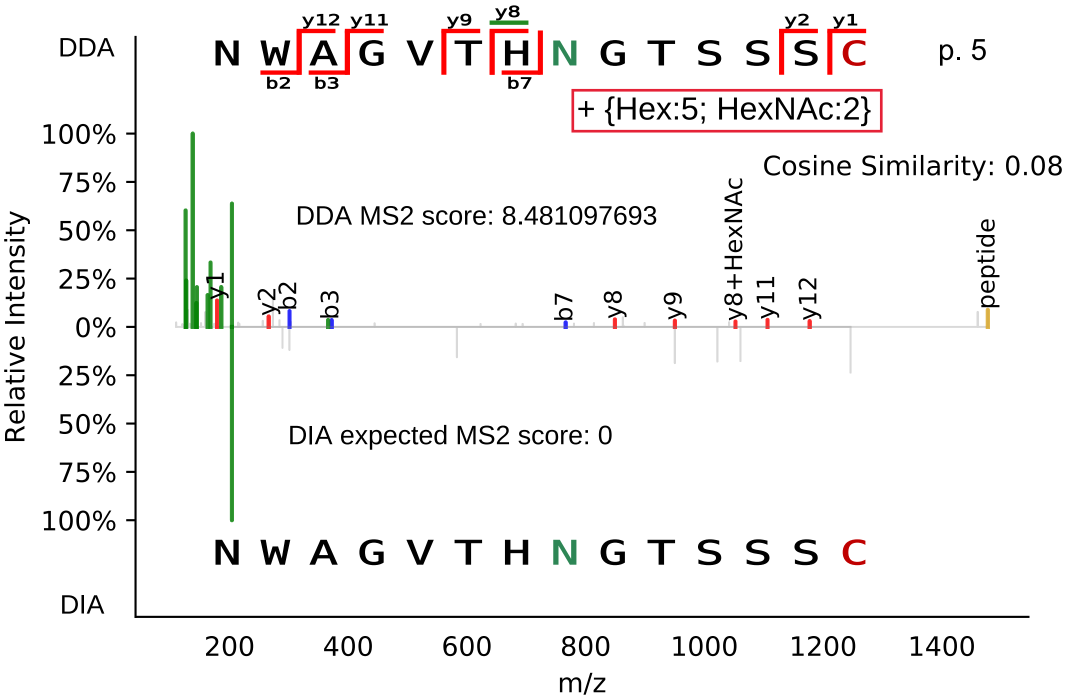

DDA
N W A G V T H N G T S S S C
y12 y11 y9 y8 y2 y1
b2 b3 b7
p. 5
+ {Hex:5; HexNAc:2}
Cosine Similarity: 0.08
DDA MS2 score: 8.481097693
DIA expected MS2 score: 0
y1 y2 b2 b3 b7 y8 y9 y8+HexNAc y11 y12 peptide
100%
75%
50%
25%
0%
Relative Intensity
N W A G V T H N G T S S S C
DIA
200
400
600
800
1000
1200
1400
m/z

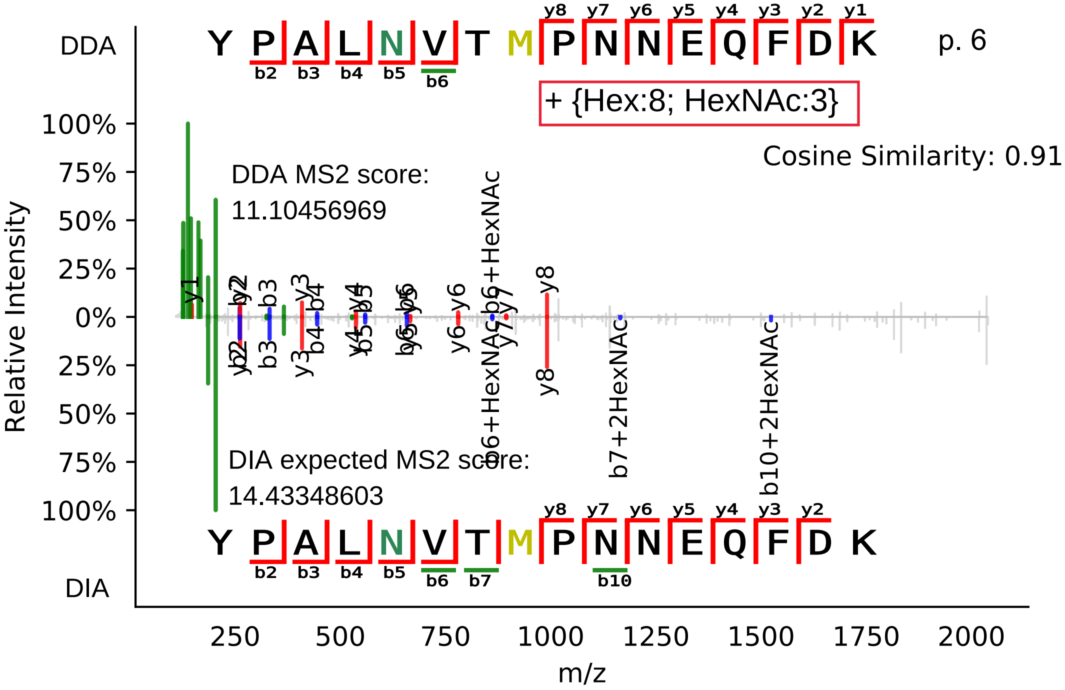

DDA
Y P A L N V T M P N N E Q F D K
y8 y7 y6 y5 y4 y3 y2 y1
b2 b3 b4 b5 b6
p. 6
+ {Hex:8; HexNAc:3}
Cosine Similarity: 0.91
DDA MS2 score:
11.10456969
DIA expected MS2 score:
14.43348603
b6+HexNAc
b7+2HexNAc
b10+2HexNAc
Y P A L N V T M P N N E Q F D K
y8 y7 y6 y5 y4 y3 y2
b2 b3 b4 b5 b6 b7 b10
DIA
Relative Intensity
100%
75%
50%
25%
0%
25%
50%
75%
100%
250
500
750
1000
1250
1500
1750
2000
m/z

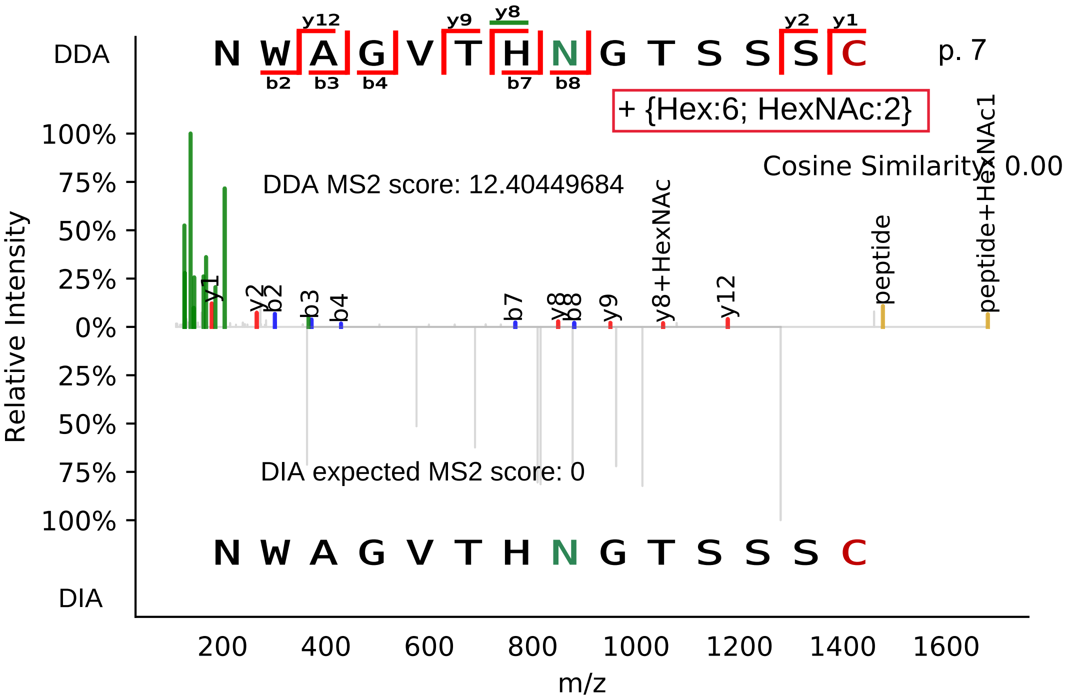

DDA
N W A G V T H N G T S S S C
y12 y9 y8 y2 y1
b2 b3 b4 b7 b8
p. 7
+ {Hex:6; HexNAc:2}
Cosine Similarity: 0.00
DDA MS2 score: 12.40449684
DIA expected MS2 score: 0
100%
75%
50%
25%
0%
25%
50%
75%
100%
Relative Intensity
y1
y2
b2
b3
b4
b7
y8
b8
y9
y8+HexNAc
y12
peptide
peptide+HexNAc1
N W A G V T H N G T S S S C
DIA
200
400
600
800
1000
1200
1400
1600
m/z

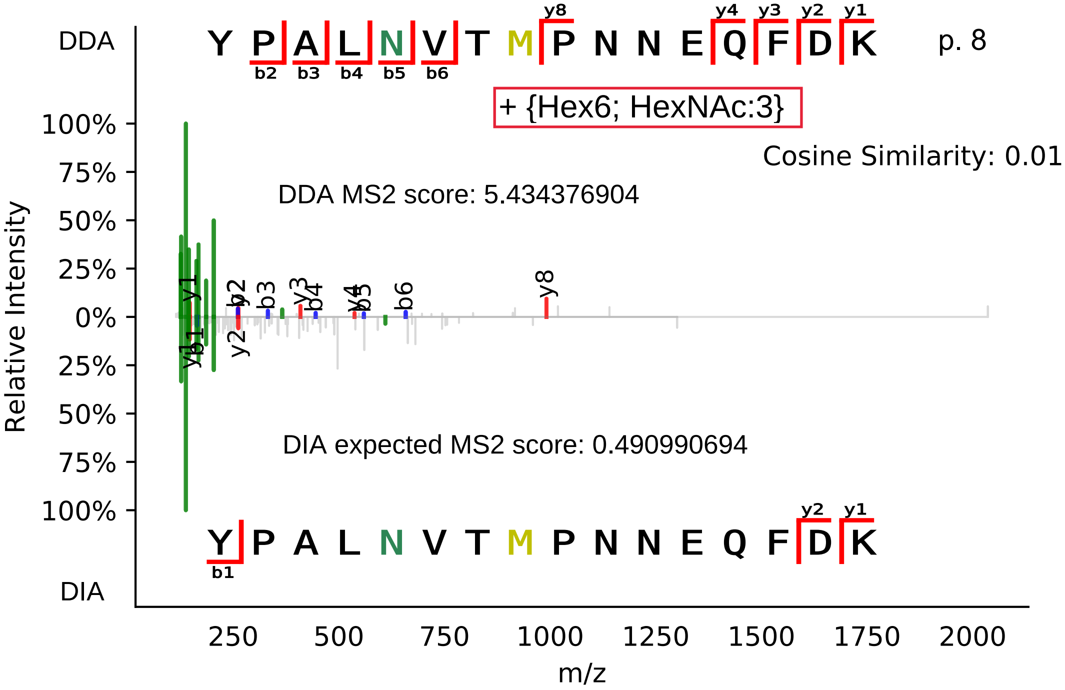
DDA
Y P A L N V T M P N N E Q F D K
y8 y4 y3 y2 y1
b2 b3 b4 b5 b6
p. 8
+ {Hex6; HexNAc:3}
Cosine Similarity: 0.01
DDA MS2 score: 5.434376904
DIA expected MS2 score: 0.490990694
Relative Intensity
100%
75%
50%
25%
0%
25%
50%
75%
100%
y1
b2
y2
b3
y3
b4
y4
b5
b6
y8
y1
b1
y2
Y P A L N V T M P N N E Q F D K
y2 y1
b1
DIA
250
500
750
1000
1250
1500
1750
2000
m/z

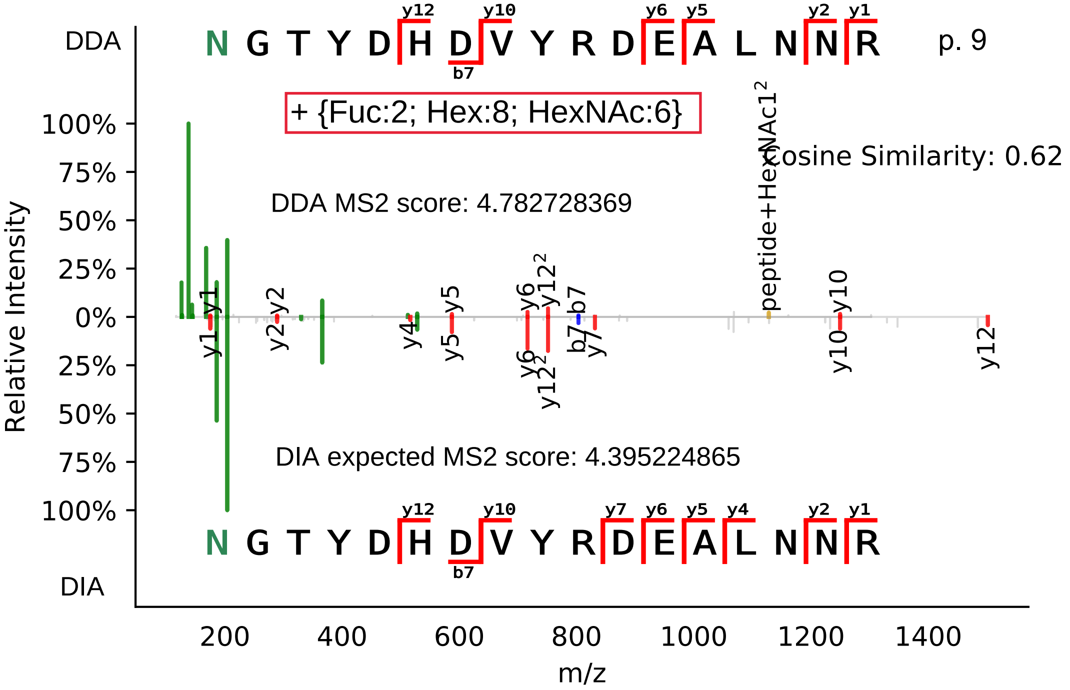

DDA
N G T Y D H D V Y R D E A L N N R
p. 9
+ {Fuc:2; Hex:8; HexNAc:6}
Cosine Similarity: 0.62
DDA MS2 score: 4.782728369
peptide+HexNAc1 2
DIA expected MS2 score: 4.395224865
DIA
N G T Y D H D V Y R D E A L N N R
Relative Intensity
100%
75%
50%
25%
0%
200
400
600
800
1000
1200
1400
m/z

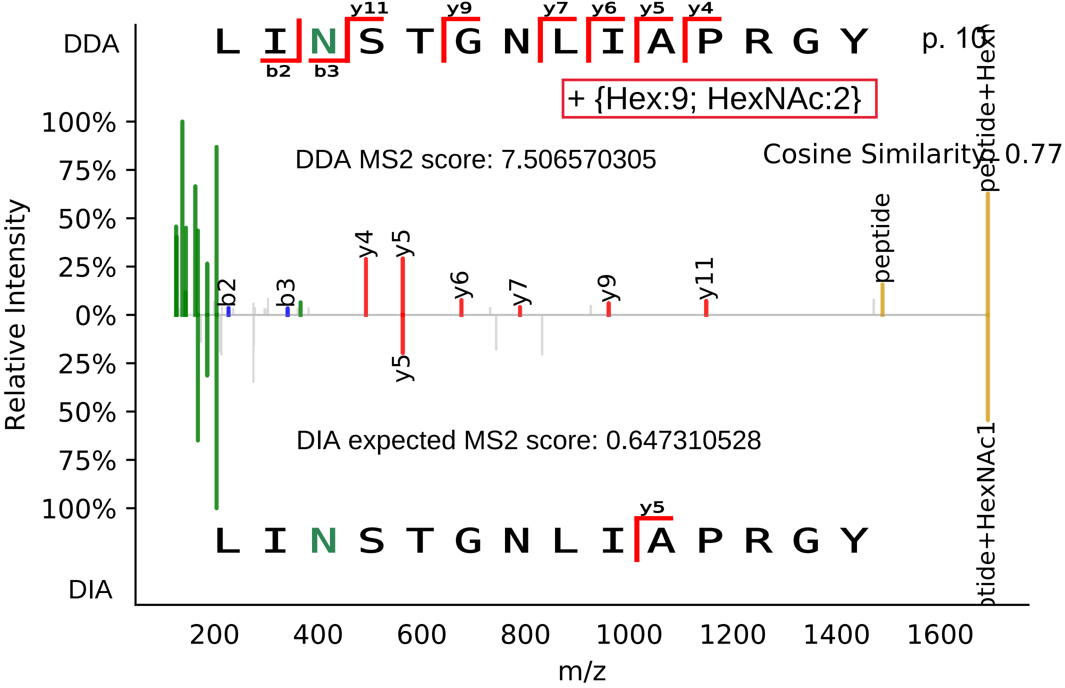

DDA
L I N S T G N L I A P R G Y
y11 y9 y7 y6 y5 y4
b2 b3
p. 10
+ {Hex:9; HexNAc:2}
DDA MS2 score: 7.506570305
Cosine Similarity: 0.77
DIA expected MS2 score: 0.647310528
Relative Intensity
100%
75%
50%
25%
0%
b2
b3
y4
y5
y6
y7
y9
y11
peptide
peptide+Hex
ptide+HexNAc1
y5
L I N S T G N L I A P R G Y
DIA
200
400
600
800
1000
1200
1400
1600
m/z

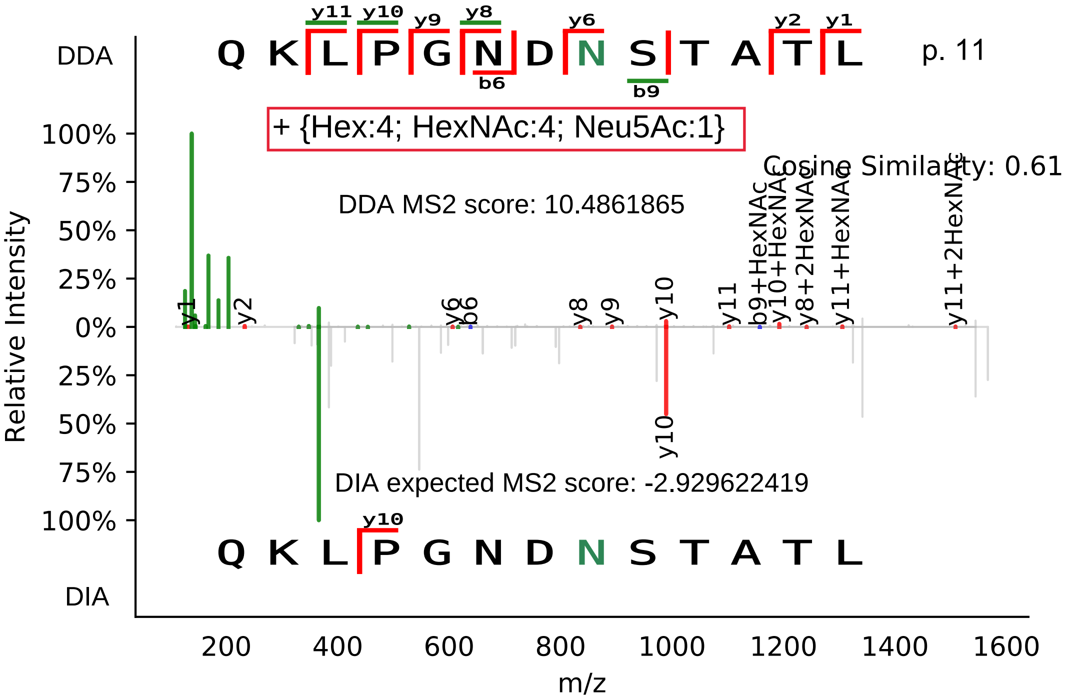
DDA
Q K L P G N D N S T A T L
y11 y10 y9 y8 y6 y2 y1
b6 b9
+ {Hex:4; HexNAc:4; Neu5Ac:1}
Cosine Similarity: 0.61
DDA MS2 score: 10.4861865
DIA expected MS2 score: -2.929622419
Relative Intensity
100%
75%
50%
25%
0%
y1 y2 y6 b6 y8 y9 y10 y11 b9+HexNAc y10+HexNAc y8+2HexNAc y11+HexNAc y11+2HexNAc
y10
y10
Q K L P G N D N S T A T L
DIA
200 400 600 800 1000 1200 1400 1600
m/z

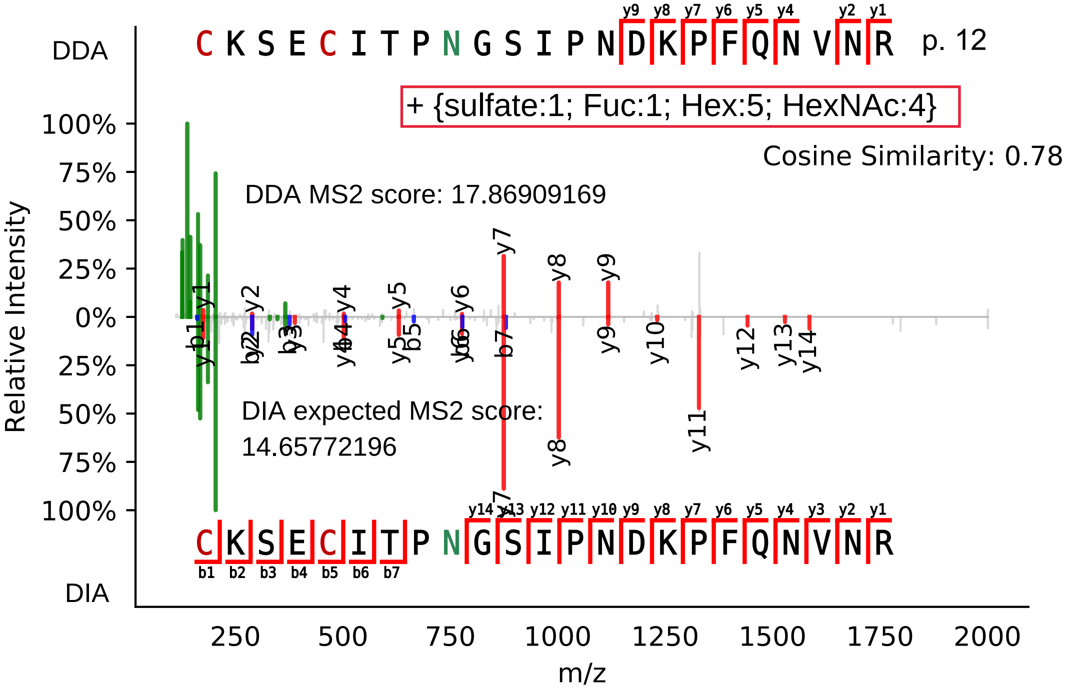
DDA
C K S E C I T P N G S I P N D K P F Q N V N R
y9 y8 y7 y6 y5 y4 y2 y1
p. 12
+ {sulfate:1; Fuc:1; Hex:5; HexNAc:4}
Cosine Similarity: 0.78
DDA MS2 score: 17.86909169
DIA expected MS2 score:
14.65772196
C K S E C I T P N G S I P N D K P F Q N V N R
y14 y13 y12 y11 y10 y9 y8 y7 y6 y5 y4 y3 y2 y1
b1 b2 b3 b4 b5 b6 b7
DIA
Relative Intensity
100%
75%
50%
25%
0%
25%
50%
75%
100%
250
500
750
1000
1250
1500
1750
2000
m/z

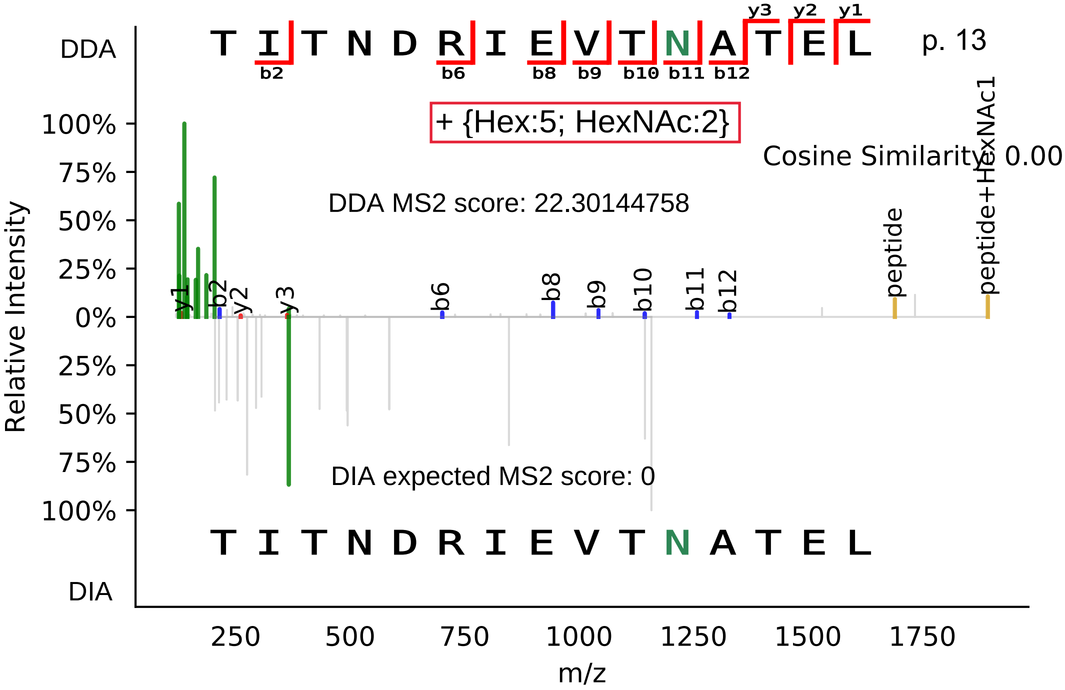

DDA
T I T N D R I E V T N A T E L
y3 y2 y1
b2 b6 b8 b9 b10 b11 b12
p. 13
+ {Hex:5; HexNAc:2}
Cosine Similarity: 0.00
DDA MS2 score: 22.30144758
DIA expected MS2 score: 0
y1 b2 y2 y3 b6 b8 b9 b10 b11 b12 peptide peptide+HexNAc1
Relative Intensity
100% 75% 50% 25% 0% 25% 50% 75% 100%
T I T N D R I E V T N A T E L
DIA
250 500 750 1000 1250 1500 1750
m/z

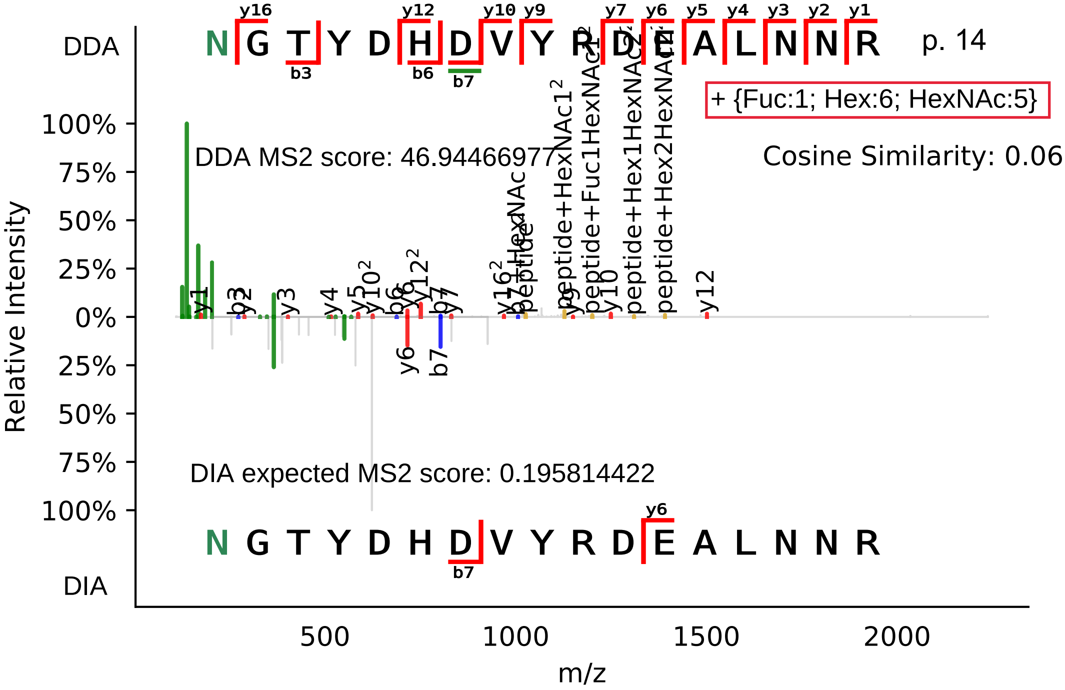

DDA
N G T Y D H D V Y R D E A L N N R
p. 14
+ {Fuc:1; Hex:6; HexNAc:5}
DDA MS2 score: 46.94466977
Cosine Similarity: 0.06
peptide+HexNAc1
peptide+Fuc1HexNAc1
peptide+Hex1HexNAc1
peptide+Hex2HexNAc1
DIA expected MS2 score: 0.195814422
Relative Intensity
m/z
DIA

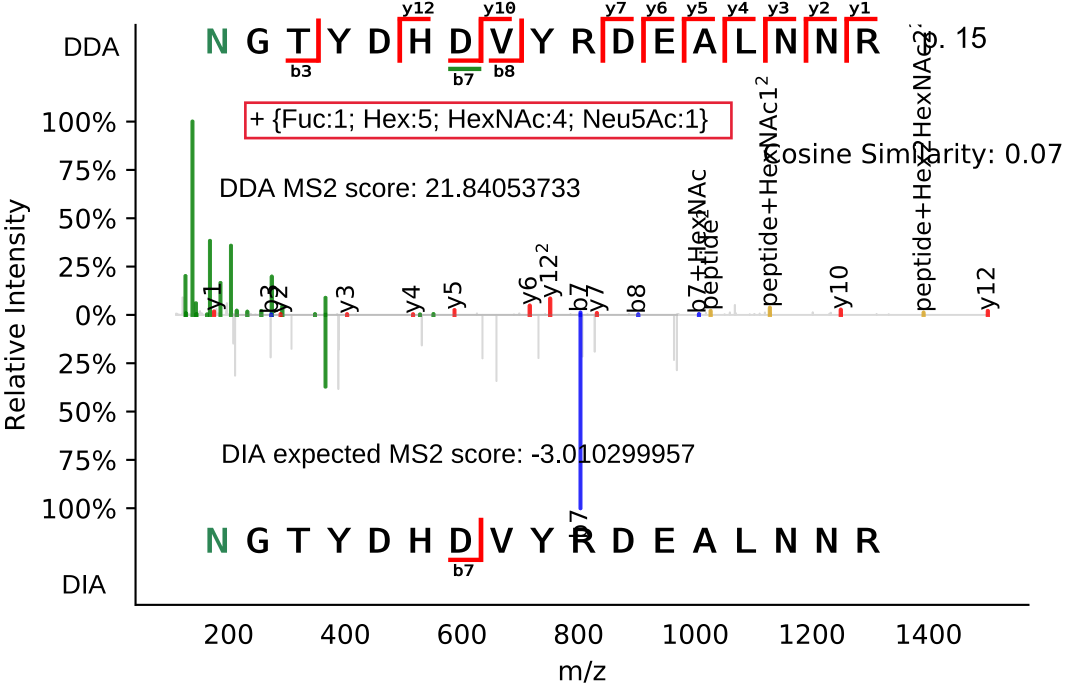

DDA
N G T Y D H D V Y R D E A L N N R
y12 y10 y7 y6 y5 y4 y3 y2 y1
b3 b7 b8
p. 15
+ {Fuc:1; Hex:5; HexNAc:4; Neu5Ac:1}
Cosine Similarity: 0.07
DDA MS2 score: 21.84053733
DIA expected MS2 score: -3.010299957
100%
75%
50%
25%
0%
25%
50%
75%
100%
Relative Intensity
y1 b3 y2 y3 y4 y5 y6 y12² b7 y7 b8 b7+HexNAc peptide² peptide+HexNAc1² y10 y12
b7
N G T Y D H D V Y R D E A L N N R
b7
DIA
200
400
600
800
1000
1200
1400
m/z

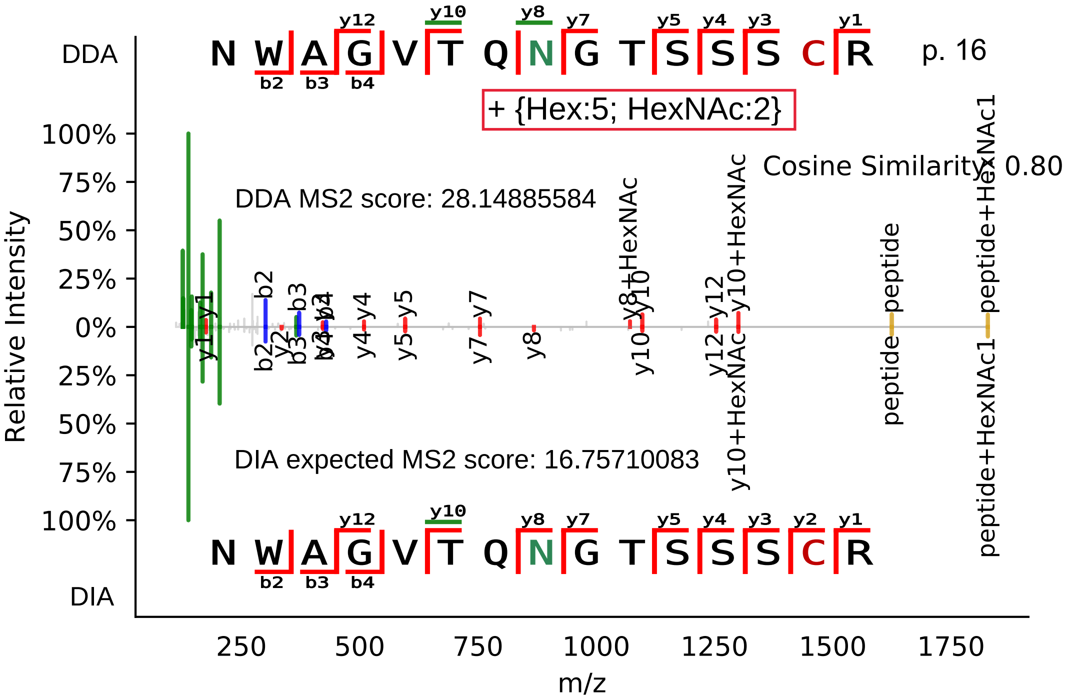
DDA
N W A G V T Q N G T S S S C R
y12 y10 y8 y7 y5 y4 y3 y1
b2 b3 b4
+ {Hex:5; HexNAc:2}
Cosine Similarity: 0.80
DDA MS2 score: 28.14885584
DIA expected MS2 score: 16.75710083
Relative Intensity
100%
75%
50%
25%
0%
y8+HexNAc
y10+HexNAc
y12
y10
peptide
peptide+HexNAc1
y10+HexNAc
DIA
N W A G V T Q N G T S S S C R
y12 y10 y8 y7 y5 y4 y3 y2 y1
250 500 750 1000 1250 1500 1750
m/z

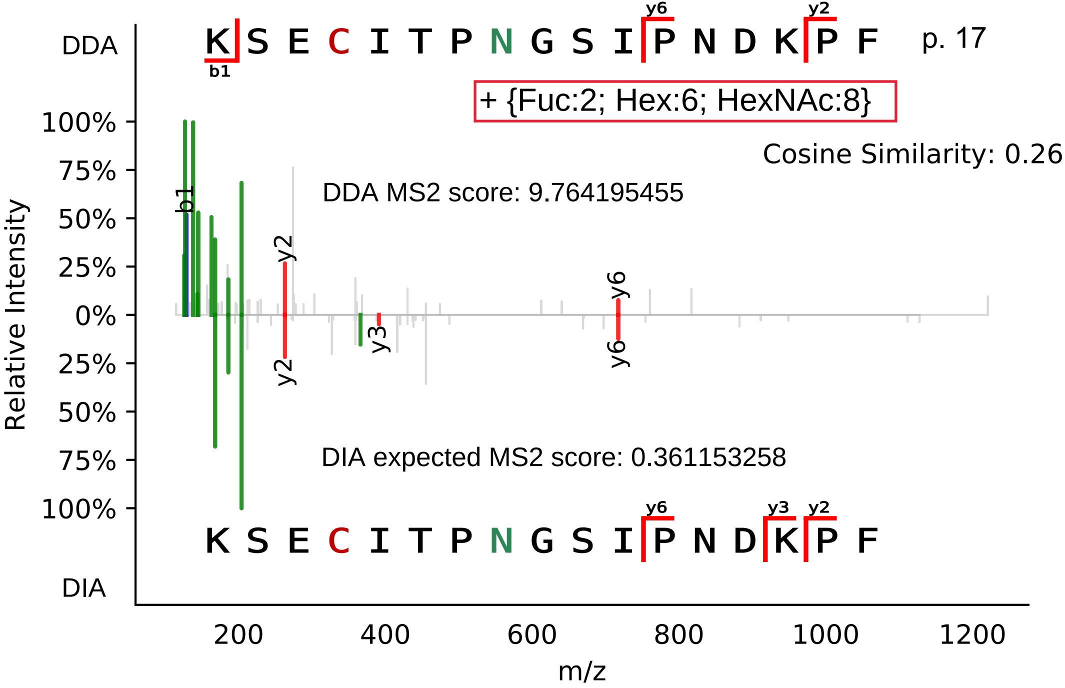
DDA
K S E C I T P N G S I P N D K P F
b1
y6
y2
+ {Fuc:2; Hex:6; HexNAc:8}
Cosine Similarity: 0.26
DDA MS2 score: 9.764195455
DIA expected MS2 score: 0.361153258
Relative Intensity
100%
75%
50%
25%
0%
25%
50%
75%
100%
b1
y2
y6
y2
y3
y6
K S E C I T P N G S I P N D K P F
y6
y3
y2
DIA
200
400
600
800
1000
1200
m/z

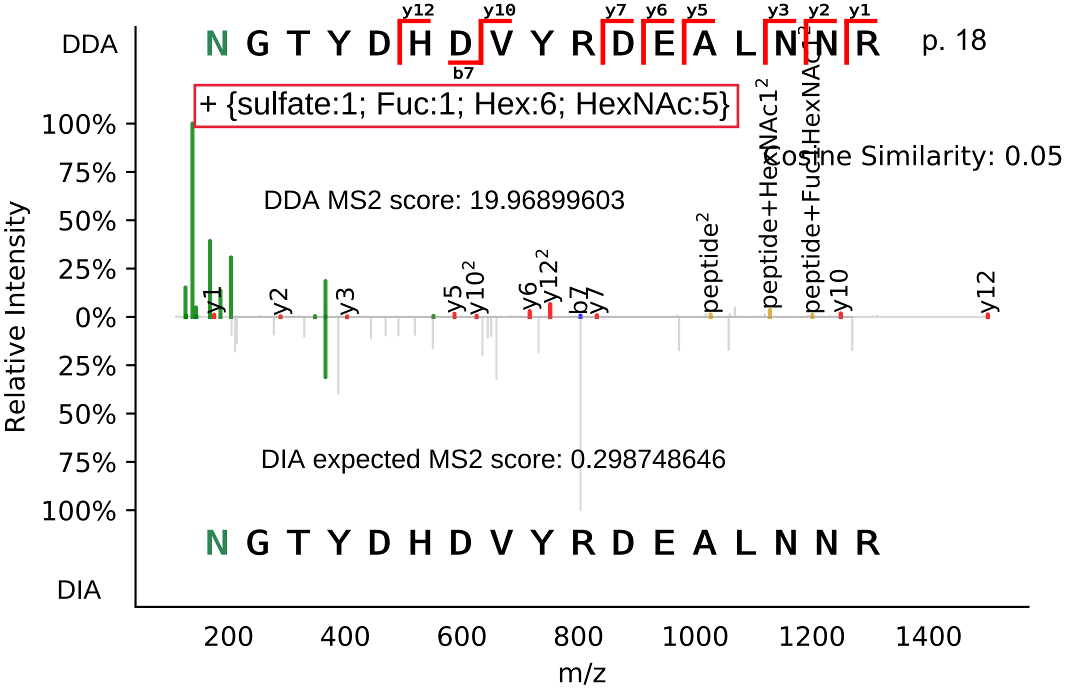
DDA
N G T Y D H D V Y R D E A L N N R
y12 y10 y7 y6 y5 y3 y2 y1
b7
+ {sulfate:1; Fuc:1; Hex:6; HexNAc:5}
Cosine Similarity: 0.05
DDA MS2 score: 19.96899603
DIA expected MS2 score: 0.298748646
Relative Intensity
100%
75%
50%
25%
0%
y1 y2 y3 y5 y10² y6 y12² b7 y7 peptide² peptide+HexNAc1² peptide+Fuc1HexNAc1² y10 y12
N G T Y D H D V Y R D E A L N N R
DIA
200 400 600 800 1000 1200 1400
m/z

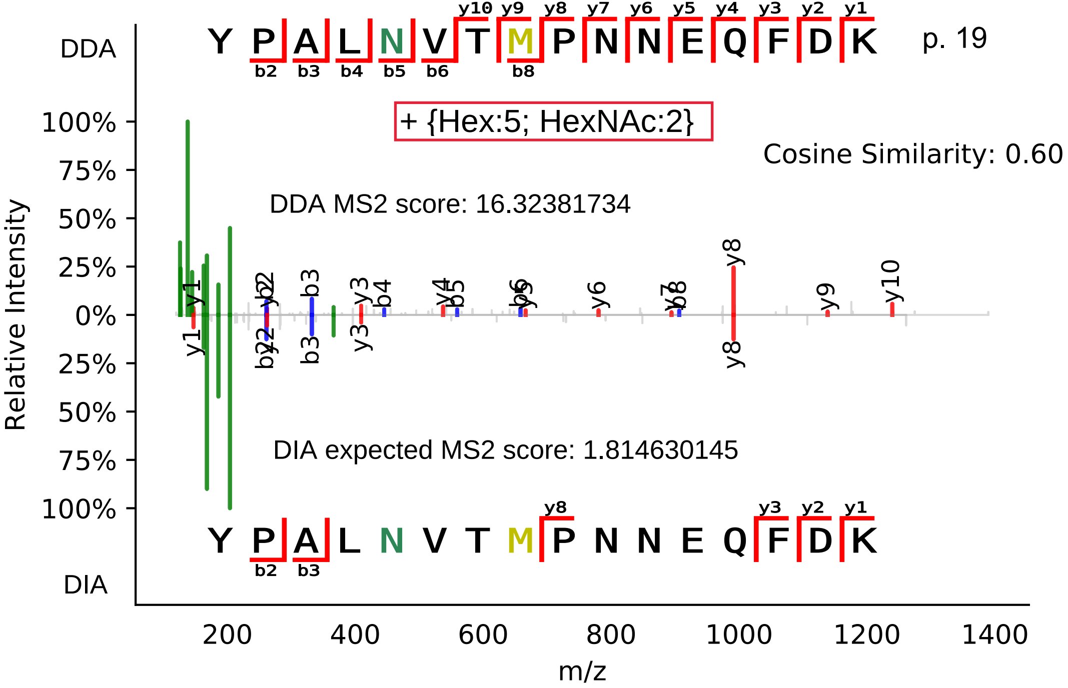
DDA
Y P A L N V T M P N N E Q F D K
y10 y9 y8 y7 y6 y5 y4 y3 y2 y1
b2 b3 b4 b5 b6 b8
+ {Hex:5; HexNAc:2}
Cosine Similarity: 0.60
DDA MS2 score: 16.32381734
DIA expected MS2 score: 1.814630145
Relative Intensity
100%
75%
50%
25%
0%
25%
50%
75%
100%
y1 b2 y2 b3 y3 b4 y4 b5 b6 y5 y6 y7 b8 y8 y9 y10
y1 b2 y2 b3 y3 y8
Y P A L N V T M P N N E Q F D K
y8 y3 y2 y1
b2 b3
DIA
200
400
600
800
1000
1200
1400
m/z

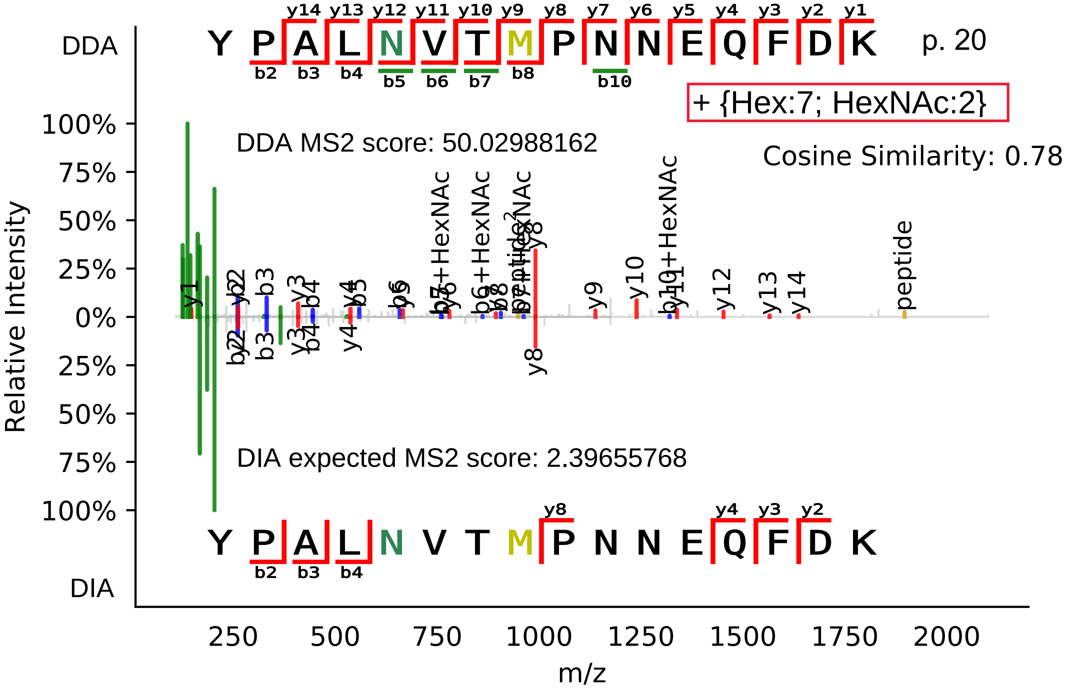

DDA
Y P A L N V T M P N N E Q F D K
y14 y13 y12 y11 y10 y9 y8 y7 y6 y5 y4 y3 y2 y1
b2 b3 b4 b5 b6 b7 b8 b10
p. 20
+ {Hex:7; HexNAc:2}
DDA MS2 score: 50.02988162
Cosine Similarity: 0.78
DIA expected MS2 score: 2.39655768
Y P A L N V T M P N N E Q F D K
y8 y4 y3 y2
b2 b3 b4
DIA
Relative Intensity
100%
75%
50%
25%
0%
25%
50%
75%
100%
250
500
750
1000
1250
1500
1750
2000
m/z

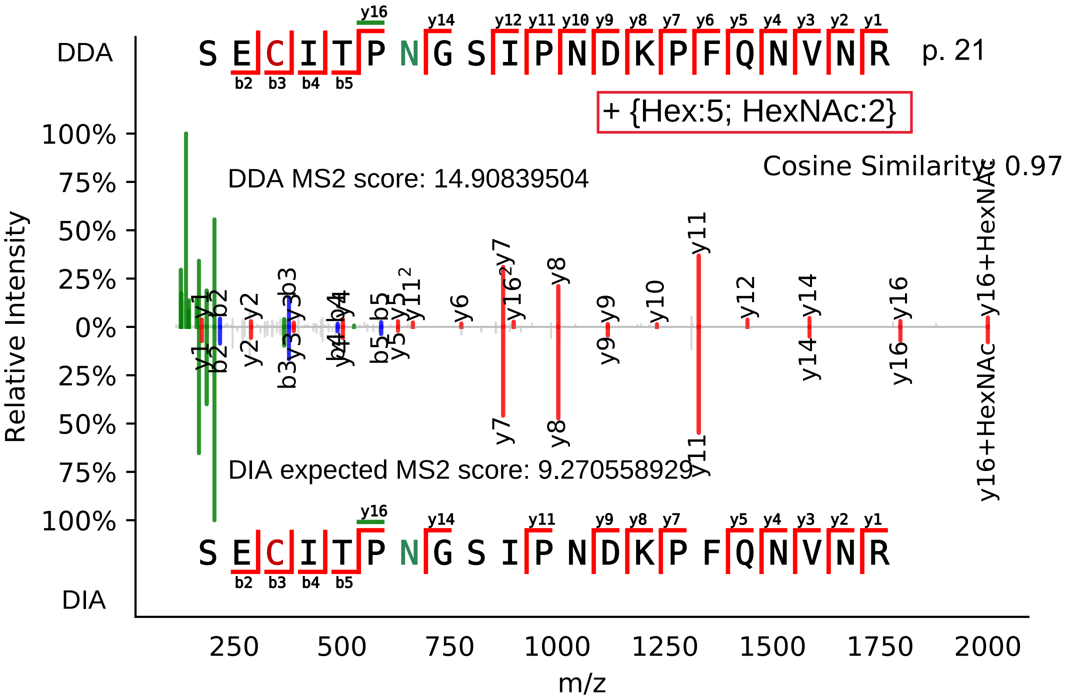
DDA
S E C I T P N G S I P N D K P F Q N V N R
y16 y14 y12 y11 y10 y9 y8 y7 y6 y5 y4 y3 y2 y1
b2 b3 b4 b5
+ {Hex:5; HexNAc:2}
Cosine Similarity: 0.97
DDA MS2 score: 14.90839504
DIA expected MS2 score: 9.270558929
y16+HexNAc
DIA
S E C I T P N G S I P N D K P F Q N V N R
y16 y14 y11 y9 y8 y7 y5 y4 y3 y2 y1
b2 b3 b4 b5
Relative Intensity
100%
75%
50%
25%
0%
m/z
250
500
750
1000
1250
1500
1750
2000
